# Healthcare disparities among anticoagulation therapies for severe COVID‐19 patients in the multi‐site VIRUS registry

**DOI:** 10.1002/jmv.26918

**Published:** 2021-03-30

**Authors:** Christian Kirkup, Colin Pawlowski, Arjun Puranik, Ian Conrad, John C. O'Horo, Dina Gomaa, Valerie M Banner‐Goodspeed, Jarrod M Mosier, Igor Borisovich Zabolotskikh, Steven K. Daugherty, Michael A. Bernstein, Howard A. Zaren, Vikas Bansal, Brian Pickering, Andrew D. Badley, Rahul Kashyap, A. J. Venkatakrishnan, Venky Soundararajan

**Affiliations:** ^1^ nference, Inc. Cambridge Massachusetts USA; ^2^ Mayo Clinic Rochester Minnesota USA; ^3^ University of Cincinnati Cincinnati Ohio USA; ^4^ Beth Israel Deaconess Medical Center Boston Massachusetts USA; ^5^ Banner University Medical Center Tucson Arizona USA; ^6^ Kuban State Medical University Krasnodar Russia; ^7^ Cox Medical Center Springfield Missouri USA; ^8^ Stamford Health Stamford Connecticut USA; ^9^ St. Joseph's Candler Health System Savannah Georgia USA

**Keywords:** biostatistics & bioinformatics, epidemiology, pandemics, social science

## Abstract

Here we analyze hospitalized andintensive care unit coronavirus disease 2019 (COVID‐19) patient outcomes from the international VIRUS registry (https://clinicaltrials.gov/ct2/show/NCT04323787). We find that COVID‐19 patients administered unfractionated heparin but not enoxaparin have a higher mortality‐rate (390 of 1012 = 39%) compared to patients administered enoxaparin but not unfractionated heparin (270 of 1939 = 14%), presenting a risk ratio of 2.79 (95% confidence interval [CI]: [2.42, 3.16]; *p* = 4.45e−52). This difference persists even after balancing on a number of covariates including demographics, comorbidities, admission diagnoses, and method of oxygenation, with an increased mortality rate on discharge from the hospital of 37% (268 of 733) for unfractionated heparin versus 22% (154 of 711) for enoxaparin, presenting a risk ratio of 1.69 (95% CI: [1.42, 2.00]; *p* = 1.5e−8). In these balanced cohorts, a number of complications occurred at an elevated rate for patients administered unfractionated heparin compared to patients administered enoxaparin, including acute kidney injury, acute cardiac injury, septic shock, and anemia. Furthermore, a higher percentage of Black/African American COVID patients (414 of 1294 [32%]) were noted to receive unfractionated heparin compared to White/Caucasian COVID patients (671 of 2644 [25%]), risk ratio 1.26 (95% CI: [1.14, 1.40]; *p* = 7.5e−5). After balancing upon available clinical covariates, this difference in anticoagulant use remained statistically significant (311 of 1047 [30%] for Black/African American vs. 263 of 1047 [25%] for White/Caucasian, *p* = .02, risk ratio 1.18; 95% CI: [1.03, 1.36]). While retrospective studies cannot suggest any causality, these findings motivate the need for follow‐up prospective research into the observed racial disparity in anticoagulant use and outcomes for severe COVID‐19 patients.

## 1 INTRODUCTION

Major complications of severe coronavirus disease 2019 (COVID‐19) include coagulopathy and cardiovascular events.^1^, ^2^, ^3^ Through the National Institutes of Health (NIH) ACTIV initiative, there are multiple ongoing research studies to evaluate the safety and effectiveness of various types and doses of anticoagulants.^4^ According to NIH Director Francis S. Collins, MD, PhD, “There is currently no standard of care for anticoagulation in hospitalized COVID‐19 patients, and there is a desperate need for clinical evidence to guide practice.” Due to the current knowledge gap in evidence‐based anticoagulant treatments for severe COVID‐19, there are many open questions on topics including: types of anticoagulant medications to prescribe, dosing for anticoagulants, indications for anticoagulant prescriptions, and prophylactic versus therapeutic use.

In this paper, we focus on which types of anticoagulant medications to prescribe for patients with severe COVID‐19. We conduct this analysis on the Society for Critical Care Medicine's (SCCM's) Viral Infection and Respiratory Illness Universal Study (VIRUS) registry,^5^ a large‐scale, international, multi‐site study of hospitalized COVID‐19 patients. While the availability of anticoagulant dosing information in the SCCM registry is relatively sparse, we are able to examine differential patient outcomes associated with whether a patient has or has not received a specific anticoagulant medication. We consider three categories of anticoagulant medications: (1) Unfractionated Heparin, (2) Enoxaparin, and (3) Other types of low molecular weight heparin (LMWH). First, we consider head‐to‐head comparisons of enoxaparin versus unfractionated heparin and enoxaparin versus other types of LMWH by constructing cohorts of hospitalized COVID patients who received one anticoagulant medication but not the other during their hospital stay for COVID‐19. For each cohort comparison, we evaluate patient outcomes including: mortality at hospital discharge, 28‐day mortality status, average hospital length of stay in days, average intensive care unit (ICU) length of stay in days, and complications during the 28‐day follow‐up period. In addition, for each comparison we repeat the analysis using propensity score matching to control for potential confounding variables including: demographics, comorbidities, evidence of infiltrates, ICU admission status, initial oxygenation method, admission diagnoses, and time in days to anticoagulant administration. Finally, we analyzed the rates of anticoagulant medication administration by race, focusing on cohorts of Black/African American and White/Caucasian patients. Similar, we used propensity score matching to construct race‐based cohorts balanced on the clinical covariates listed previously, and we report patient outcomes for both the original and the propensity‐matched race‐stratified cohorts.

## 2 METHODS

### 2.1 Study design

The SCCM's Discovery VIRUS: COVID‐19 Registry is composed of data collected from patients hospitalized for COVID‐19. As of January 4, 2021, the total size of the study population is 29,950 patients reported by 192 hospitals across 20 countries. While a portion of sites report data for each day in the hospital for each patient, emphasis is placed on capturing data at key events in the treatment process. These include the day of admission to the hospital, the first 3 days in the hospital, and first day of admission to the ICU (if admitted) as well as outcomes measures like the duration of stay in the hospital and the ICU (if admitted) and the 28‐day survival status. Data completeness of the features is variable depending on the frequency of updates from the sites. Data for the registry is collected via REDCap and can be automatically filled from a site's EHR data.

Features reported include comorbidities listed in the VIRUS questionnaire (obesity, diabetes, hypertension, etc.), complications (acute kidney injury, deep vein thrombosis, coagulopathy, etc.), medications prescribed in hospital (antibacterials, anticoagulants, statins, etc.) as well as more refined medication features within a category (antivirals: remdesivir, ritonavir, lopinavir, etc.). Other features collected for each patient include hospital length of stay, ICU Length of stay, height, weight, etc. For the purposes of length of stay analysis, hospital length of stay or ICU length of stay durations greater than 90 days are excluded based on the premise that these values may be reported in error and reflect the duration of stay in hours rather than in days.

Prior studies suggest that enoxaparin may be more efficacious than unfractionated heparin in the treatment of conditions like acute coronary syndromes^6^ and these are two most frequently administered anticoagulants (Table S1). Thus, we compare the outcomes of patients taking enoxaparin and heparin by constructing two cohorts: (i) patients who were administered enoxaparin but not unfractionated heparin and (ii) patients who were administered unfractionated heparin but not enoxaparin. The cohort sizes were 1814 and 887, respectively. Statistical tests were applied to 21 outcomes (with Benjamini–Hochberg procedure applied to account for the problem of multiple comparisons; details below). Mortality at hospital discharge was the primary outcome of interest. Outcomes that were compared include (1) mortality at hospital discharge, (2) mortality at 28 days, (3) admission to ICU (within 28 days of hospitalization), (4) length of stay in ICU (among alive patients), (5) length of stay in hospital (among alive patients), and the following 16 complications: (6) acute cardiac injury, (7) acute kidney injury, (8) anemia, (9) bacteremia, (10) bacterial pneumonia, (11) cardiac arrest, (12) cardiac arrhythmia, (13) co‐ or secondary infection, (14) congestive heart failure, (15) deep vein thrombosis, (16) hyperglycemia, (17) liver dysfunction, (18) pleural effusions, (19) ARDS, (20) septic shock, (21) stroke or cerebrovascular incident. The diagnostic criteria available are outlined in Table S2.

To account for potentially confounding variables, we performed propensity score matching to balance covariates between the two cohorts. The statistical tests for differences in outcomes were repeated on the matched cohorts. The covariates which were balanced include demographics, comorbidities, and various features on admission. Further detail on the procedure, including a listing of covariates used, is below. The code to process the raw data files was written in R v3.6.1. The code to perform the statistical analyses was written in Python v3.7.7, using the scikit‐learn package v0.23.2 to train the logistic regression models for the propensity score matching step. In Table S3, we show the data completeness for the clinical covariates that we used for matching. Most covariates have close to full completeness (over 90%), with the exception of the “evidence of infiltrates” covariate, which has roughly 80% data completeness. For this field, missing values were imputed to be the mean of other values of the field within the treatment group.

### 2.2 Statistical methods

For each of the cohort comparisons, we ran a series of statistical significance tests to compare across each of the patient outcome variables of interest. For categorical outcome variables (e.g. mortality status, complications), we report the proportion of patients in each cohort that have the outcome variable, the relative risk (ratio of proportions for each cohort), 95% confidence interval (CI) for the relative risk, and *χ* ^2^ *p*‐value. The function stats. chi2_contingency from the SciPy package in Python was used to compute the *χ* ^2^ *p*‐values. For continuous outcome variables (e.g., hospital/ICU length of stay), we report the mean and standard deviation of the variable in each cohort, along with the *p* value from a two‐sided Mann–Whitney test (stats. mannwhitneyu from SciPy) comparing the two cohorts. Finally, we apply the Benjamini–Hochberg correction to adjust *p* values for multiple comparisons.

### 2.3 Propensity score matching

To control for potential confounding factors which may explain differences in patient outcomes between the enoxaparin and unfractionated heparin cohorts, we used propensity score matching to balance the cohorts.^7^ First, propensity scores for each of the patients in the two cohorts were computed by fitting a logistic regression model as a function of the clinical covariates (listed below). Next, patients from the enoxaparin and unfractionated heparin cohorts were matched using a 1:1 matching ratio and a heuristic caliper of 0.1 x pooled standard deviation,^8^ allowing for drops. Before matching, there were 2120 patients in the enoxaparin cohort (administered enoxaparin but not unfractionated heparin), and there were 1076 patients in the unfractionated heparin cohort (administered unfractionated heparin but not enoxaparin). From these two cohorts, 778 matched pairs were found, and statistical analyses were run on the final matched cohorts. Here is the full list of covariates that were considered for the propensity score matching step:

- Demographics: age, gender, race, ethnicity.
- Comorbidities: pre‐existing conditions, including: (1) asthma, (2) blood loss anemia, (3) cardiac arrhythmias, (4) chronic kidney disease, (5) chronic dialysis, (6) chronic pulmonary disease, (7) coagulopathy, (8) congestive heart failure, (9) coronary artery disease, (10) dementia, (11) depression, (12) diabetes, (13) dyslipidemia/hyperlipidemia, (14) HIV/AIDS or other immunosuppression, (15) hematologic malignancy, (16) hepatitis B, (17) hepatitis C, (18) history of solid organ or bone marrow transplant, (19) hypertension, (20) hypothyroidism, (21) iron deficiency anemia, (22) liver disease, (23) malnutrition, (24) metastatic cancer, (25) obstructive sleep apnea/home CPAP/bilevel positive airway pressure (BiPAP) use, (26) obesity, (27) paralysis, (28) peptic ulcer disease excluding bleeding, (29) psychosis, (30) pulmonary circulation disorder, (31) rheumatoid arthritis/collagen vascular disease, (32) solid tumor without metastasis, (33) stroke or other neurological disorders, (34) substance use disorder, (35) valvular heart disease, (36) venous thromboembolism (deep vein thrombosis/pulmonary embolism)
- In ICU on admission to hospital
- Admission diagnoses: Conditions which are present upon admission to hospital for COVID‐19, including: (1) acute respiratory distress syndrome (ARDS), (2) non‐ARDS acute hypoxic respiratory failure, (3) acute liver injury, (4) acute myocardial infarction, (5) acute renal failure/injury (with or without hemofiltration) (6) bacteremia, (7) bacterial pneumonia, (8) cardiac arrest, (9) cardiac arrhythmias (atrial fibrillation, heart block, torsades des point, ventricular tachycardia), (10) congestive heart failure/cardiomyopathy, (11) delirium/encephalopathy, (12) disseminated intravascular coagulation, (13) gastrointestinal hemorrhage, (14) hyperglycemia, (15) hypoglycemia, (16) meningitis/encephalitis, (17) myocarditis, (18) pleural effusion, (19) pneumothorax, (20) rhabdomyolysis/myositis, (21) seizure, (22) sepsis, (23) shock, (24) stroke.
- Evidence of infiltrates via X‐ray or CT scan
- Oxygenation‐related features on admission: Supplemental oxygenation method on day of admission, among: (1) invasive mechanical ventilation, (2) noninvasive ventilation (CPAP or BIPAP), (3) high flow nasal cannula, (4) bag mask oxygen therapy, (5) non‐rebreather mask oxygen therapy, (6) nasal cannula, (7) miscellaneous other form of oxygenation.
- Day of anticoagulant administration: First day that the patient received the anticoagulant of interest (unfractionated heparin, enoxaparin, or other LMWH), relative to the day of hospital admission for COVID‐19.

The same propensity score matching procedure was done with enoxaparin versus other low molecular weight heparin in place of enoxaparin versus unfractionated heparin. Propensity score matching was also applied to balance covariates between the Black/African American and White/Caucasian patient cohorts; the “outcome” compared in this case was the rate of administration of each anticoagulant. All of the same covariates (except race and day of anticoagulant administration) listed above were used in this balancing.

## 3 RESULTS

**Figure 1:**
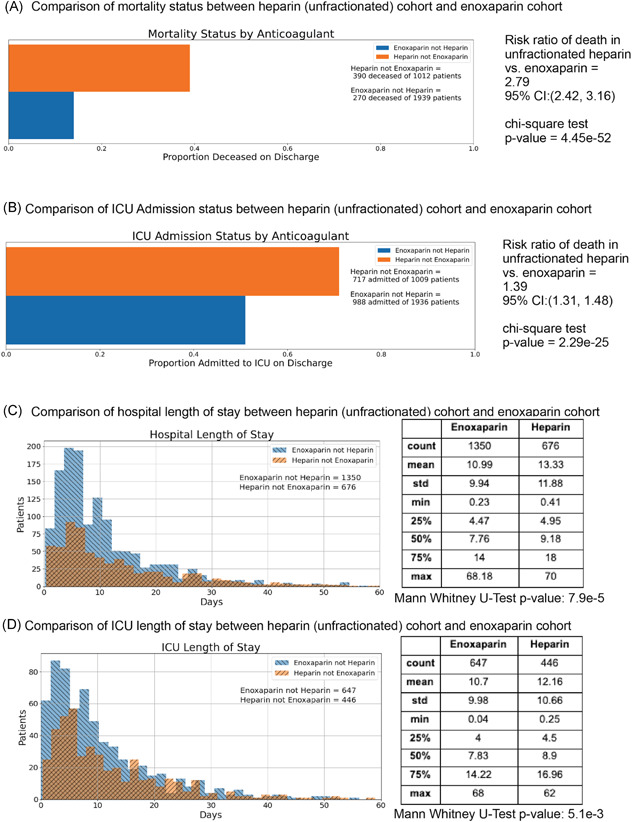
Comparison of outcomes between unfractionated heparin and enoxaparin patient cohorts (unadjusted). (A) Bar charts show a comparison of mortality status at discharge from the hospital between patient cohorts receiving enoxaparin but not heparin (blue) or unfractionated heparin but not enoxaparin (orange) during hospitalization. (B) Bar charts show a comparison of mortality status at discharge from the hospital between patient cohorts receiving enoxaparin but not unfractionated heparin (blue) or unfractionated heparin but not enoxaparin (orange) during hospitalization. (C) Histograms show intensive care unit (ICU) Length of Stay in days for cohorts of alive patients who received enoxaparin but not unfractionated heparin (blue) and reported a length of stay in the ICU and alive patients who received unfractionated heparin but not enoxaparin (orange) and reported a length of stay in the ICU. (D) Histograms show hospital Length of Stay in days for cohorts of alive patients who received enoxaparin but not unfractionated heparin (blue) and reported a length of stay in the ICU and alive patients who received unfractionated heparin but not enoxaparin (orange) and reported a length of stay in the hospital. CI, confidence interval

In Figure 1, we present the mortality rate and ICU admission rate for patients in the SCCM VIRUS registry^5^ with outcomes data available. Among the 29,950 patients in the VIRUS registry at the time of the study, hospital discharge status was available for 16,859 patients, of which 2894 (17%) were deceased at discharge. For patients that were administered unfractionated heparin but not enoxaparin, hospital discharge status was available for 1012 patients, and 390 (39%) were deceased at discharge. For patients that were administered enoxaparin but not unfractionated heparin, hospital discharge status was available for 1939 patients, of which 270 (14%) were deceased at discharge. Comparing the mortality outcomes (unadjusted), patients in the heparin cohort have a higher mortality rate compared to those in the enoxaparin cohort (risk ratio: 2.79; 95% CI: [2.42, 3.16]; *p* = 4.45e−52) (Figure 1A). For patients that were administered unfractionated heparin but not enoxaparin, ICU admission status was available for 1009 patients, of which 717 (71%) were admitted to the ICU. Similarly, for patients that were administered enoxaparin but not unfractionated heparin, ICU admission status was available for 1936 patients, of which 988 (51%) were admitted to the ICU. Comparing the ICU admission status (unadjusted), patients administered unfractionated heparin had a higher rate of admission to the ICU compared to patients administered enoxaparin (risk ratio: 1.39; 95% CI: [1.31, 1.48]; *p* = 2.29e−25) (Figure 1B).

Next, we compared the average lengths of stay in the ICU and hospital for the two cohorts. Here, we restricted the analysis to only patients that were alive at discharge. Among patients with length of stay information available, the average length of stay in the hospital was shorter for the enoxaparin patients (mean hospital duration: 10.99 days; 1350 patients) compared to the unfractionated heparin patients (mean hospital duration: 13.33 days; 676 patients) (Figure 1C). For patients admitted to the ICU, the length of stay in the ICU was also shorter for enoxaparin patients (mean ICU duration: 10.70 days; 647 patients) compared to unfractured heparin patients (mean ICU duration: 12.16 days; 446 patients) (Figure 1D). While the difference on average hospital length of stay is statistically significant (Mann–Whitney *p* = 7.9e−5), the difference on average ICU length of stay is also statistically significant (Mann–Whitney *p* = 5.1e−3).

**Figure 2:**
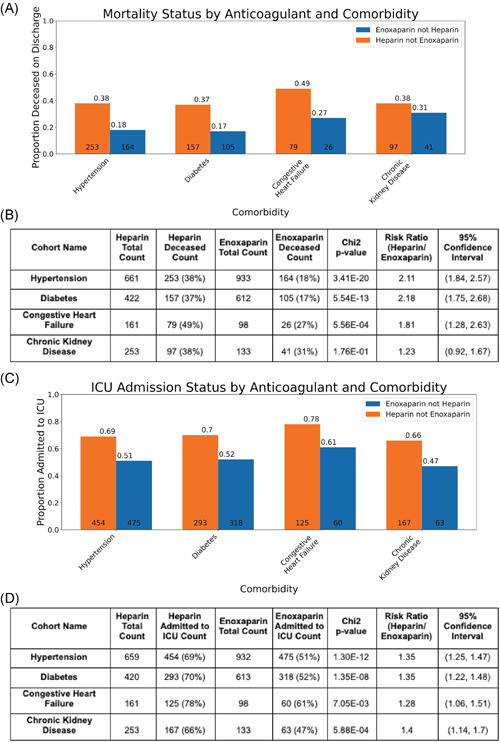
Comparison of outcomes between unfractionated heparin and enoxaparin patient cohorts in patients also reporting comorbidities. Bar charts show a comparison of Mortality Status at discharge from the hospital and status of admission to the ICU for two cohorts—patients receiving enoxaparin and reporting a comorbidity of interest (blue), and patients receiving heparin and reporting a comorbidity of interest (orange). Comorbidities include—hypertension, diabetes, chronic kidney disease and congestive heart failure. Statistics for these plots are included in the corresponding tables. CI, confidence interval; ICU, intemsive care unit

In Figure 2, we present the mortality rate and ICU admission rate for patients with different comorbidities: diabetes, hypertension, chronic kidney disease, and congestive heart failure. We observe that for the subgroups of patients with diabetes, hypertension, and congestive heart failure, patients administered enoxaparin have significantly lower rates of ICU admission and death compared to patients administered unfractionated heparin. For patients with chronic kidney disease, the difference in ICU admission rates between the unfractionated heparin and enoxaparin cohorts is statistically significant (risk ratio: 1.4; 95% CI: [1.14, 1.7]; *p* = 5.88e−04), however, the difference in mortality status is not statistically significant (risk ratio: 1.23; 95% CI: [0.92, 1.67], *p* = .18).

Next, we perform propensity score matching to control for a wide array of confounding factors simultaneously. The clinical characteristics of the matched and original unfractionated heparin and enoxaparin cohorts are shown in Table 1. Most covariates (including demographics, comorbidities, and admission diagnoses) appear well‐matched.

**Table 1:** Covariate balancing results for enoxaparin and unfractionated heparin cohorts

**Clinical covariate**	**Unfractionated heparin cohort (matched)**	**Enoxaparin cohort (matched)**	**Unfractionated heparin cohort (original)**	**Enoxaparin cohort (original)**
Total number of patients	778	778	1076	2120
Age in years (standard deviation)	62 (18)	60.1 (19.1)	63.3 (17.3)	63.7 (323)
Sex				
– Male	463/777 (60%)	460 (59%)	651/1075 (61%)	1173 (55%)
Race				
– Asian	78 (10%)	64 (8.2%)	99 (9.2%)	236 (11%)
– Black/African American	190 (24%)	192 (25%)	301 (28%)	448 (21%)
– Other	122 (16%)	113 (15%)	162 (15%)	423 (20%)
– White/Caucasian	386 (50%)	409 (53%)	511 (47%)	1012 (48%)
Ethnicity				1201/2119 (57%)
– Hispanic	456/777 (59%)	466 (60%)	632/1075 (59%)	
Pregnant	12 (1.5%)	12 (1.5%)	13 (1.2%)	25 (1.2%)
Comorbidities				
– Asthma	54 (6.9%)	57 (7.3%)	77 (7.2%)	184 (8.7%)
– Cancer	46 (5.9%)	54 (6.9%)	73 (6.8%)	142 (6.7%)
– Cardiac arrhythmias	63 (8.1%)	62 (8%)	109 (10%)	108 (5.1%)
– Chronic dialysis	20 (2.6%)	10 (1.3%)	88 (8.2%)	10 (0.47%)
– Chronic kidney disease	115 (15%)	120 (15%)	268 (25%)	142 (6.7%)
– Chronic pulmonary disease	66 (8.5%)	73 (9.4%)	100 (9.3%)	164 (7.7%)
– Congestive heart failure	75 (9.6%)	70 (9%)	164 (15%)	108 (5.1%)
– Coronary artery disease	120 (15%)	122 (16%)	205 (19%)	192 (9.1%)
– Dementia	48 (6.2%)	59 (7.6%)	77 (7.2%)	91 (4.3%)
– Depression	82 (11%)	84 (11%)	112 (10%)	195 (9.2%)
– Diabetes	286 (37%)	266 (34%)	448 (42%)	668 (32%)
– Hypertension	463 (60%)	456 (59%)	701 (65%)	1026 (48%)
– Hypothyroidism	60 (7.7%)	70 (9%)	91 (8.5%)	144 (6.8%)
– Obesity	157 (20%)	154 (20%)	215 (20%)	470 (22%)
– Obstructive sleep apnea with home CPAP/BIPAP use	54 (6.9%)	60 (7.7%)	86 (8%)	153 (7.2%)
– Stroke or other neurologic disorders	67 (8.6%)	66 (8.5%)	107 (9.9%)	133 (6.3%)
Evidence of infiltrates via X‐ray or CT scan	451/582 (77%)	501/622 (81%)	643/814 (79%)	1328/1717 (77%)
ICU admission on first day of hospitalization	345 (44%)	349 (45%)	530 (49%)	702 (33%)
Oxygenation method on first day of hospitalization				
– Any	535 (69%)	535 (69%)	771 (72%)	1455 (69%)
– High flow nasal cannula	62 (8%)	77 (9.9%)	78 (7.2%)	177 (8.3%)
– Invasive mechanical ventilation	185 (24%)	187 (24%)	340 (32%)	221 (10%)
– Nasal cannula	288 (37%)	275 (35%)	361 (34%)	986 (47%)
– Noninvasive mechanicalventilation (CPAP/BIPAP)	29 (3.7%)	29 (3.7%)	35 (3.3%)	93 (4.4%)
– Bag mask	19 (2.4%)	21 (2.7%)	25 (2.3%)	63 (3%)
– Non‐rebreather mask	75 (9.6%)	77 (9.9%)	103 (9.6%)	154 (7.3%)
Admission diagnosis				
– Acute hypoxic respiratory failure	356 (46%)	360 (46%)	510 (47%)	1005 (47%)
(non‐ARDS)				
– Acute kidney injury	152 (20%)	139 (18%)	266 (25%)	204 (9.6%)
– ARDS	106 (14%)	107 (14%)	160 (15%)	203 (9.6%)
– Bacterial pneumonia	88 (11%)	92 (12%)	124 (12%)	210 (9.9%)
– Cardiac arrest	13 (1.7%)	8 (1%)	30 (2.8%)	15 (0.71%)
– Cardiac arrhythmias	0 (0)	0 (0)	0 (0)	0 (0)
– Congestive heart failure	17 (2.2%)	20 (2.6%)	50 (4.6%)	26 (1.2%)
– Delirium/encephalopathy	65 (8.4%)	76 (9.8%)	112 (10%)	98 (4.6%)
– Hyperglycemia	56 (7.2%)	51 (6.6%)	89 (8.3%)	155 (7.3%)
– Sepsis	112 (14%)	116 (15%)	186 (17%)	282 (13%)
– Shock	42 (5.4%)	43 (5.5%)	90 (8.4%)	73 (3.4%)
– Stroke	14 (1.8%)	8 (1%)	32 (3%)	9 (0.42%)
Average time (days) for first anticoagulant administration relative to hospital admission (enoxaparin or heparin).	1 (2.33)	0.925 (1.85)	1.04 (2.45)	0.786 (1.53)
Propensity score for enoxaparin versus heparin treatment (standard deviation)	0.471 (0.196)	0.479 (0.2)	0.377 (0.231)	0.623 (0.186)

*Note:* Summary of patient characteristics for matched and original cohorts of hospitalized COVID‐19 patients who have taken either: unfractionated heparin or enoxaparin (but not both). For numeric variables, such as age and first date of anticoagulant administration, the mean value for each cohort is shown with standard deviation in parentheses. For categorical variables, such as race and ethnicity, patient counts are shown with the percentage of each cohort in parentheses. Denominators are shown when the variable has substantial missing data.

Abbreviations: ARDS, acute respiratory distress syndrome; BIPAP, bilevel positive airway pressure; COVID‐19, coronavirus disease 2019; CPAP, continuous positive airway pressure.

Of the 778 patients in the matched heparin cohort, mortality status at discharge was available for 733, of which 268 (37%) were deceased on discharge; in the matched enoxaparin cohort, information was available for 711 patients of which 154 (22%) were deceased on discharge (Table 2). This difference in mortality rates upon discharge was statistically significant (risk ratio: 1.69; 95% CI: [1.42, 2.00]; adjusted *p* = 1.5e−8). The mortality rates reported at 28‐days for both cohorts were consistent with the mortality rates reported upon hospital discharge, and differences in rates between the two cohorts were similarly statistically significant. Differences between the two cohorts in the average hospital and ICU length of stays were not statistically significant after matching.

**Table 2:** Comparison of patient outcomes for enoxaparin and unfractionated heparin cohorts

**Outcome variable**	**Heparin cohort (matched) (*n* = 778)**	**Enoxaparin cohort (matched) (*n* = 778)**	**BH‐adjusted *p* value**	**Relative risk (95% confidence interval [CI]) Heparin versus enoxaparin**
Number of patients with reported outcomes				
– Mortality status at hospital discharge (alive or deceased)	733	711		
– Mortality status at 28 days (alive or deceased)	463	528
– ICU admission	732	714		
– Hospital length of stay	328	393
– ICU length of stay	159	199		
– Complications during hospitalization	758	742
Mortality at hospital discharge	268/733 (37%)	154/711 (22%)	1.5e−8	1.69 (1.42, 2.00)
Mortality at 28 days	44/463 (9.5%)	12/528 (2.3%)	1.3e−5	4.18 (2.19, 7.51)
ICU admission during hospitalization	481/732 (66%)	399/714 (56%)	8.8e−4	1.18 (1.08, 1.28)
Hospital length of stay (days)	12.7 (12.2)	11.7 (10.3)	.88	
ICU length of stay (days)	12.3 (11.7)	10.9 (10.3)	.47	
Complications during hospitalization				
– Acute cardiac injury	39 (5.1%)	18 (2.4%)	.03	2.12 (1.21, 3.60)
– Acute kidney injury	280 (37%)	182 (25%)	2.9E−06	1.51 (1.29, 1.76)
– ARDS	224 (30%)	200 (27%)	.47	1.10 (0.93, 1.29)
– Anemia	101 (13%)	70 (9.4%)	.07	1.41 (1.06, 1.88)
– Bacteremia	49 (6.5%)	35 (4.7%)	.35	1.37 (0.90, 2.08)
– Bacterial pneumonia	94 (12%)	95 (13%)	.92	0.97 (0.74, 1.26)
– Cardiac arrest	74 (9.8%)	65 (8.8%)	.73	1.11 (0.81, 1.53)
– Cardiac arrhythmia	54 (7.1%)	50 (6.7%)	.92	1.06 (0.73, 1.53)
– Co‐ or secondary infection	59 (7.8%)	53 (7.1%)	.87	1.09 (0.76, 1.55)
– Congestive heart failure	28 (3.7%)	14 (1.9%)	.12	1.96 (1.03, 3.59)
– Deep vein thrombosis	23 (3%)	21 (2.8%)	.94	1.07 (0.60, 1.90)
– Hyperglycemia	76 (10%)	103 (14%)	.07	0.72 (0.55, 0.96)
– Liver dysfunction	59 (7.8%)	47 (6.3%)	.47	1.23 (0.85, 1.77)
– Pleural effusions	36 (4.7%)	24 (3.2%)	.35	1.47 (0.88, 2.41)
– Septic shock	144 (19%)	95 (13%)	.01	1.48 (1.17, 1.88)
– Stroke/cerebrovascular incident	19 (2.5%)	11 (1.5%)	.40	1.69 (0.81, 3.42)
– Viral pneumonitis	111 (15%)	119 (16%)	.68	0.91 (0.72, 1.16)

*Note:* Summary of clinical outcomes for matched cohorts of hospitalized COVID‐19 patients who have taken either unfractionated heparin or enoxaparin (but not both). For categorical variables, such as mortality status and complications, patient counts are shown with the percentage of each cohort in parentheses. Only patients with reported outcomes in each cohort are used to determine the percentages. For numeric variables, such as hospital and ICU length of stay, the mean value for each cohort is shown with standard deviation in parentheses. In addition, Benjamini–Hochberg adjusted *p* values are shown for the statistical tests comparing the outcome variables for the matched enoxaparin and Heparin cohorts; relative risk of outcomes (heparin relative to enoxaparin) are shown as well, along with 95% CI.

Abbreviations: ARDS, acute respiratory distress syndrome; COVID‐19, coronavirus disease 2019; ICU, intensive care unit.

Information on complications that occurred after admission was available for 758 of 778 patients in the matched heparin cohort, and for 742 of 778 patients in the matched enoxaparin cohort. Complications that occurred at a significantly higher rate in the matched heparin cohort compared to the matched enoxaparin cohort include: acute kidney injury (280 of 758 [37%] vs. 182 of 742 [25%], respectively, adjusted *p* = 2.9e−6), acute cardiac injury (39 of 758 [5.1%] vs. 18 of 742 [2.4%], respectively, adjusted *p* = .03), and septic shock (144 of 758 [19%] vs. 95 of 742 [13%], respectively, adjusted *p* = .01) (Table 2).

We also conducted an equivalent analysis comparing enoxaparin versus other types of LMWH. The matching table is shown in Table 3. Of the 851 patients in this matched other LMWH cohort, mortality status at discharge was available for 778 patients, of which 263 (34%) were deceased on discharge. Of the 851 patients in the matched other LMWH cohort, information was available for 779 patients, of which 170 (22%) were deceased on discharge (risk ratio: 1.55; 95% CI: [1.31, 1.83]; adjusted *p* = 2.0e−6) (Table 4). Complications which show statistically significant differences between the other LMWH matched cohort and the enoxaparin matched cohort include: Acute cardiac injury (52 of 804 [6.5%] vs. 25 of 810 [3.1%], respectively; adjusted *p* = 7.9e−3), Bacterial pneumonia (68 of 804 [8.5%] vs. 124 of 810 [15%]; adjusted *p* = 2.2e−4), Liver dysfunction (42 of 804 [5.2%] vs. 82 of 810 [10%]; adjusted *p* = 1.7e−3), and Viral pneumonitis (50 of 804 [6.2%] vs. 148 of 810 [18%]; adjusted *p* = 6.2e−12).

**Table 3:** Covariate balancing results for enoxaparin and other low molecular weight heparin (LMWH) cohorts

**Clinical covariate**	**Other LMWH cohort (matched)**	**Enoxaparin cohort (matched)**	**LMWH cohort (original)**	**Enoxaparin cohort (original)**
Total number of patients	851	851	964	2442
Age in years (standard deviation)	60.7 (19.7)	57.8 (18.9)	61.1 (19.2)	63.2 (301)
Sex				
– Male	490/850 (58%)	479 (56%)	563/963 (58%)	1394 (57%)
Race				
– Asian	83 (9.8%)	91 (11%)	87 (9%)	266 (11%)
– Black/African American	190 (22%)	201 (24%)	208 (22%)	550 (23%)
– Other	141 (17%)	112 (13%)	159 (16%)	487 (20%)
– White/Caucasian	433 (51%)	447 (53%)	506 (52%)	1137 (47%)
Ethnicity			424/961 (44%)	1411/2440 (58%)
– Hispanic	397/848 (47%)	432 (51%)		
Pregnant	12 (1.4%)	9 (1.1%)	12 (1.2%)	30 (1.2%)
Comorbidities				
– Asthma	59 (6.9%)	76 (8.9%)	64 (6.6%)	223 (9.1%)
– Cancer	62 (7.3%)	60 (7.1%)	67 (7%)	164 (6.7%)
– Cardiac arrhythmias	59 (6.9%)	66 (7.8%)	78 (8.1%)	129 (5.3%)
– Chronic dialysis	17 (2%)	13 (1.5%)	29 (3%)	18 (0.74%)
– Chronic kidney disease	80 (9.4%)	78 (9.2%)	103 (11%)	181 (7.4%)
– Chronic pulmonary disease	55 (6.5%)	64 (7.5%)	60 (6.2%)	198 (8.1%)
– Congestive heart failure	91 (11%)	88 (10%)	141 (15%)	137 (5.6%)
– Coronary artery disease	109 (13%)	108 (13%)	143 (15%)	234 (9.6%)
– Dementia	40 (4.7%)	37 (4.3%)	43 (4.5%)	116 (4.8%)
– Depression	61 (7.2%)	64 (7.5%)	66 (6.8%)	237 (9.7%)
– Diabetes	264 (31%)	260 (31%)	295 (31%)	804 (33%)
– Hypertension	425 (50%)	440 (52%)	500 (52%)	1216 (50%)
– Hypothyroidism	61 (7.2%)	55 (6.5%)	71 (7.4%)	172 (7%)
– Obesity	147 (17%)	158 (19%)	160 (17%)	554 (23%)
– Obstructive sleep apnea with home CPAP/BIPAP use	47 (5.5%)	49 (5.8%)	48 (5%)	182 (7.5%)
– Stroke or other neurologic disorders	65 (7.6%)	63 (7.4%)	83 (8.6%)	155 (6.3%)
Evidence of infiltrates via X‐ray or CT scan	394/490 (80%)	569/687 (83%)	432/530 (82%)	1588/2017 (79%)
ICU admission on first day of hospitalization	382 (45%)	412 (48%)	461 (48%)	840 (34%)
Oxygenation method on first day of hospitalization				
– Any	624 (73%)	635 (75%)	717 (74%)	1715 (70%)
– High flow nasal cannula	105 (12%)	113 (13%)	125 (13%)	205 (8.4%)
– Invasive mechanical ventilation	169 (20%)	185 (22%)	218 (23%)	310 (13%)
– Nasal cannula	313 (37%)	321 (38%)	330 (34%)	1151 (47%)
– Noninvasive mechanical ventilation (CPAP/BIPAP)	43 (5.1%)	51 (6%)	46 (4.8%)	105 (4.3%)
– Bag mask	48 (5.6%)	43 (5.1%)	58 (6%)	64 (2.6%)
– Non‐rebreather mask	100 (12%)	103 (12%)	115 (12%)	192 (7.9%)
Admission diagnosis				
– Acute hypoxic respiratory failure (non‐ARDS)	454 (53%)	456 (54%)	512 (53%)	1224 (50%)
– Acute kidney injury	90 (11%)	109 (13%)	99 (10%)	292 (12%)
– ARDS	143 (17%)	162 (19%)	180 (19%)	239 (9.8%)
– Bacterial pneumonia	90 (11%)	107 (13%)	98 (10%)	241 (9.9%)
– Cardiac arrest	14 (1.6%)	11 (1.3%)	18 (1.9%)	18 (0.74%)
– Cardiac arrhythmias	0 (0)	0 (0)	0 (0)	0 (0)
– Congestive heart failure	25 (2.9%)	23 (2.7%)	36 (3.7%)	32 (1.3%)
– Delirium/encephalopathy	47 (5.5%)	41 (4.8%)	64 (6.6%)	130 (5.3%)
– Hyperglycemia	59 (6.9%)	59 (6.9%)	62 (6.4%)	193 (7.9%)
– Sepsis	108 (13%)	115 (14%)	116 (12%)	366 (15%)
– Shock	35 (4.1%)	49 (5.8%)	37 (3.8%)	113 (4.6%)
– Stroke	6 (0.71%)	6 (0.71%)	25 (2.6%)	9 (0.37%)
Day of first anticoagulant administration relative to hospital admission (enoxaparin or heparin)	0.827 (2.07)	0.937 (2.09)	0.803 (2.01)	1 (2.06)
Propensity score for enoxaparin versus LMWH treatment (standard deviation)	0.456 (0.172)	0.462 (0.174)	0.423 (0.187)	0.577 (0.164)

*Note:* Summary of patient characteristics for matched and original cohorts of hospitalized COVID‐19 patients who have taken either enoxaparin or some other LMWH. For numeric variables, such as age and first date of anticoagulant administration, the mean value for each cohort is shown with standard deviation in parentheses. For categorical variables, such as race and ethnicity, patient counts are shown with the percentage of each cohort in parentheses. Denominators are shown for the covariates which have some missing data.

Abbreviations: ARDS, acute respiratory distress syndrome; BIPAP, bilevel positive airway pressure; COVID‐19, coronavirus disease 2019; CPAP, continuous positive airway pressure; CT, computed tomography; ICU, intensive care unit.

**Table 4:** Comparison of patient outcomes for enoxaparin and other LMWH cohorts

**Outcome variable**	**Other LMWH cohort (matched) (*n* = 851)**	**Enoxaparin cohort (matched) (*n* = 851)**	**BH‐adjusted *p* value**	**Relative risk (95% CI), other LMWH versus enoxaparin**
Number of patients with reported outcomes				
– Mortality status at hospital discharge (alive or deceased)	778	779		
– Mortality status at 28 days (alive or deceased)	496	589
– ICU admission	779	781		
– Hospital length of stay	411	404
– ICU length of stay	256	240		
– Complications during hospitalization	804	810
Mortality at hospital discharge	263/778 (34%)	170/779 (22%)	2.0e–06	1.55 (1.31, 1.83)
Mortality at 28 days	8/496 (1.6%)	16/589 (2.7%)	.42	0.59 (0.27, 1.39)
ICU admission during hospitalization	525/779 (67%)	502/781 (64%)	.34	1.05 (0.98, 1.13)
Hospital length of stay (days)	13.9 (9.94)	13 (10.6)	.06	
ICU length of stay (days)	9.42 (8.49)	10.5 (9.03)	.42	
Complications during hospitalization				
– Acute cardiac injury	52 (6.5%)	25 (3.1%)	7.9e−03	2.10 (1.31, 3.30)
– Acute kidney injury	163 (20%)	204 (25%)	.06	0.80 (0.67, 0.97)
– ARDS	262 (33%)	235 (29%)	.24	1.12 (0.97, 1.30)
– Anemia	74 (9.2%)	93 (11%)	.26	0.80 (0.60, 1.07)
– Bacteremia	31 (3.9%)	46 (5.7%)	.22	0.68 (0.44, 1.06)
– Bacterial pneumonia	68 (8.5%)	124 (15%)	2.2e−04	0.55 (0.42, 0.73)
– Cardiac arrest	79 (9.8%)	75 (9.3%)	.84	1.06 (0.79, 1.43)
– Cardiac arrhythmia	35 (4.4%)	53 (6.5%)	.17	0.67 (0.44, 1.01)
– Co‐ or secondary infection	47 (5.8%)	55 (6.8%)	.61	0.86 (0.59, 1.25)
– Congestive heart failure	13 (1.6%)	19 (2.3%)	.5	0.69 (0.35, 1.39)
– Deep vein thrombosis	17 (2.1%)	22 (2.7%)	.62	0.78 (0.42, 1.45)
– Hyperglycemia	73 (9.1%)	121 (15%)	1.7e−03	0.61 (0.46, 0.80)
– Liver dysfunction	42 (5.2%)	82 (10%)	1.7e−03	0.52 (0.36, 0.74)
– Pleural effusions	22 (2.7%)	24 (3%)	.9	0.92 (0.53, 1.63)
– Septic shock	108 (13%)	133 (16%)	.22	0.82 (0.65, 1.03)
– Stroke/cerebrovascular incident	12 (1.5%)	14 (1.7%)	.9	0.86 (0.41, 1.84)
– Viral pneumonitis	50 (6.2%)	148 (18%)	6.2e−12	0.34 (0.25, 0.46)

*Note:* Summary of clinical outcomes for matched cohorts of hospitalized COVID‐19 patients who have taken either enoxaparin or other LMWH. For categorical variables, such as mortality status and complications, patient counts are shown with the percentage of each cohort in parentheses. Only patients with reported outcomes in each cohort are used to determine the percentages. For numeric variables, such as hospital and ICU length of stay, the mean value for each cohort is shown with standard deviation in parentheses. In addition, Benjamini–Hochberg adjusted *p* values are shown for the statistical tests comparing the outcome variables for the matched enoxaparin and Heparin cohorts.

Abbreviations: ARDS, acute respiratory distress syndrome; CI, confidence interval; COVID‐19, coronavirus disease 2019; ICU, intensive care unit; LMWH, low molecular weight heparin.

We also examined whether there were any race‐based differences in the administration of enoxaparin and unfractionated heparin. The cohorts of interest were Black/African American patients with anticoagulant information available (*n* = 1294) and White/Caucasian patients with anticoagulant information available (*n* = 2644). Propensity score matching was performed, and the clinical characteristics of the matched and original cohorts are shown in Table 5. We observe that the clinical covariates are well‐balanced for the matched cohorts. Rates of administration of unfractionated heparin, enoxaparin, and other LMWH medications for the original and matched cohorts are shown in Tables 6 and 7, respectively.

**Table 5:** Covariate balancing results for race‐based cohorts

**Clinical covariate**	**Black/African American cohort (matched)**	**White/Caucasian cohort (matched)**	**Black/African American cohort (original)**	**White/Caucasian cohort (original)**
Total number of patients	1047	1047	1294	2644
Age in years (standard deviation)	55.2 (21.1)	56.4 (23.4)	55.6 (20.7)	59 (22.5)
Sex				
– Male	566 (54%)	575 (55%)	682 (53%)	1512 (57%)
Ethnicity				
– Hispanic	889 (85%)	902 (86%)	1136 (88%)	1567 (59%)
Pregnant	15 (1.4%)	7 (0.7%)	15 (1.1%)	42 (1.6%)
Comorbidities				
– Asthma	117 (11%)	109 (10%)	157 (12%)	192 (7.3%)
– Cancer	67 (6.4%)	56 (5.3%)	80 (6.2%)	239 (9%)
– Cardiac arrhythmias	66 (6.3%)	62 (5.9%)	83 (6.4%)	308 (12%)
– Chronic dialysis	33 (3.2%)	32 (3.1%)	50 (3.9%)	53 (2%)
Chronic kidney disease	154 (15%)	154 (15%)	227 (18%)	329 (12%)
– Chronic pulmonary disease	86 (8.2%)	97 (9.3%)	109 (8.4%)	257 (9.7%)
– Congestive heart failure	112 (11%)	103 (9.8%)	158 (12%)	283 (11%)
– Coronary artery disease	125 (12%)	128 (12%)	152 (12%)	403 (15%)
– Dementia	75 (7.2%)	69 (6.6%)	85 (6.6%)	180 (6.8%)
– Depression	95 (9.1%)	92 (8.8%)	105 (8.1%)	311 (12%)
– Diabetes	350 (33%)	367 (35%)	491 (38%)	739 (28%)
– Hypertension	602 (57%)	610 (58%)	815 (63%)	1301 (49%)
– Hypothyroidism	62 (5.9%)	61 (5.8%)	62 (4.8%)	258 (9.8%)
– Obesity	243 (23%)	265 (25%)	327 (25%)	511 (19%)
– ʹObstructive sleep apnea with home CPAP/BIPAP use	86 (8.2%)	103 (9.8%)	118 (9.1%)	213 (8.1%)
– Stroke or other neurologic disorders	103 (9.8%)	97 (9.3%)	126 (9.7%)	234 (8.9%)
Evidence of infiltrates via X‐ray or CT scan	637/884 (72%)	575/794 (72%)	809/1099 (74%)	1331/1823 (73%)
ICU admission on first day of hospitalization	341 (33%)	340 (32%)	405 (31%)	950 (36%)
Oxygenation method on first day of hospitalization				
– Any	632 (60%)	658 (63%)	780 (60%)	1710 (65%)
– High flow nasal cannula	100 (9.6%)	100 (9.6%)	128 (9.9%)	182 (6.9%)
– Invasive mechanical ventilation	134 (13%)	125 (12%)	168 (13%)	388 (15%)
– Nasal cannula	406 (39%)	426 (41%)	490 (38%)	1104 (42%)
– Noninvasive mechanical ventilation (CPAP/BIPAP)	27 (2.6%)	30 (2.9%)	30 (2.3%)	89 (3.4%)
– Bag mask	19 (1.8%)	16 (1.5%)	20 (1.5%)	78 (3%)
– Non‐rebreather mask	67 (6.4%)	76 (7.3%)	81 (6.3%)	193 (7.3%)
Admission diagnosis				
– Acute hypoxic respiratory failure (non‐ARDS)	436 (42%)	435 (42%)	545 (42%)	1241 (47%)
– Acute kidney injury	183 (17%)	189 (18%)	301 (23%)	304 (11%)
– ARDS	80 (7.6%)	87 (8.3%)	114 (8.8%)	188 (7.1%)
– Bacterial pneumonia	103 (9.8%)	106 (10%)	134 (10%)	247 (9.3%)
– Cardiac arrest	11 (1.1%)	5 (0.48%)	16 (1.2%)	11 (0.42%)
– Cardiac arrhythmias	0 (0)	0 (0)	0 (0)	0 (0)
– Congestive heart failure	34 (3.2%)	43 (4.1%)	52 (4%)	74 (2.8%)
– Delirium/encephalopathy	82 (7.8%)	77 (7.4%)	123 (9.5%)	165 (6.2%)
– Hyperglycemia	71 (6.8%)	62 (5.9%)	108 (8.3%)	124 (4.7%)
– Shock	181 (17%)	170 (16%)	258 (20%)	340 (13%)
– Stroke	64 (6.1%)	61 (5.8%)	94 (7.3%)	108 (4.1%)
10 (0.96%)	9 (0.86%)	10 (0.77%)	46 (1.7%)
Propensity score for white versus black subpopulation (standard deviation)	0.577 (0.192)	0.568 (0.186)	0.626 (0.202)	0.374 (0.225)

*Note:* Summary of patient characteristics for matched and original cohorts of Black/African American and White/Caucasian hospitalized COVID‐19 patients. For numeric variables, such as age and first date of anticoagulant administration, the mean value for each cohort is shown with standard deviation in parentheses. For categorical variables, such as race and ethnicity, patient counts are shown with the percentage of each cohort in parentheses.

Abbreviations: ARDS, acute respiratory distress syndrome; BIPAP, bilevel positive airway pressure; COVID‐19, coronavirus disease 2019; CPAP, continuous positive airway pressure; ICU, intensive care unit.

**Table 6:** Anticoagulant administration rates by race among unmatched cohorts

**Anticoagulant administered**	**Black/African American cohort (*n* = 1294)**	**White/Caucasian cohort (*n* = 2644)**	***χ* ^2^ test *p* value**	**BH‐corrected *p* value**	**Relative risk (95% CI)**
Enoxaparin	553 (43%)	1112 (42%)	.71	.71	1.02 (0.94, 1.10)
Unfractionated heparin	414 (32%)	671 (25%)	1.5E−05	7.5E−05	1.26 (1.14, 1.40)
Other low molecular weight heparin	226 (17%)	534 (20%)	.05	.08	0.86 (0.75, 1.00)
Enoxaparin but not unfractionated heparin	422 (33%)	924 (35%)	.16	.20	0.93 (0.85, 1.03)
Unfractionated heparin but not enoxaparin	283 (22%)	483 (18%)	.01	.02	1.20 (1.05, 1.36)

*Note:* Rates of anticoagulant administration among unmatched Black/African American and White/Caucasian cohorts of hospitalized COVID‐19 patients.

Abbreviations: CI, confidence interval; COVID‐19, coronavirus disease 2019.

**Table 7:** Anticoagulant administration rates by race among matched cohorts

**Anticoagulant administered**	**Black/African American cohort (matched) (*n* = 1047)**	**White/Caucasian cohort (matched) (*n* = 1047)**	***χ* ^2^ test, *p* value**	**BH‐corrected, *p* value**	**Relative risk (95% CI)**
Enoxaparin	453 (43%)	442 (42%)	.66	.82	1.02 (0.93, 1.13)
Unfractionated heparin	311 (30%)	263 (25%)	.02	.11	1.18 (1.03, 1.36)
Other low molecular weight heparin	186 (18%)	189 (18%)	.91	.91	0.98 (0.82, 1.18)
Enoxaparin but not unfractionated heparin	347 (33%)	369 (35%)	.33	.72	0.94 (0.84, 1.06)
Unfractionated heparin but not enoxaparin	205 (20%)	190 (18%)	.43	.72	1.08 (0.90, 1.29)

*Note:* Rates ofanticoagulant administration among matched Black/African American and White/Caucasiancohorts of hospitalized COVID‐19 patients.

Abbreviations: CI, confidence interval; COVID‐19, coronavirus disease 2019.

Looking at the original unmatched cohorts, Black/African American patients had significantly higher rates of administration of unfractionated heparin compared to White/Caucasian patients (414 of 1294 [32%] vs. 671 of 2644 [25%], respectively; adjusted *p* = 7.5e−05) (Table 6). After matching, this difference in unfractionated heparin use is not statistically significant (311 of 1047 [30%] for Black/African American patients vs. 263 of 1047 [25%] for White/Caucasian patients; adjusted *p* = .11) (Table 7). On the other hand, enoxaparin and other low molecular weight heparins are administered at similar rates in the unmatched and matched cohorts (Tables 6 and 7). Finally, the proportion of patients which took exclusively either enoxaparin or unfractionated heparin are similar for the Black/African American and White/Caucasian cohorts.

## 4 DISCUSSION

Prior work has shown that anticoagulant treatments and prophylaxis are associated with improved outcomes for COVID‐19 patients.^9^, ^10^ In particular, there is evidence to suggest that low molecular weight heparin can be used to effectively treat COVID‐19 patients with coagulopathy.^11^ This retrospective analysis suggests that enoxaparin, a particular form of low molecular weight heparin, shows promise as an anticoagulant therapy for severe COVID‐19, compared to both unfractionated heparin and other low molecular weight heparin therapies. These findings are consistent with a retrospective study on electronic health records from the Mayo Clinic which has found that enoxaparin is associated with lower rates of thrombotic events, kidney injury, and mortality in comparison with unfractionated heparin.^12^ However, this study goes beyond the previous analysis by leveraging the massive SCCM VIRUS data registry of hospitalized COVID‐19 patients from multiple sites around the world. As a result, we find that there are additional complications which are enriched at a statistically significant level in the unfractionated heparin cohort compared to the enoxaparin cohort, including septic shock and anemia.

There are several limitations of this study. While we have longitudinal data from the registry on daily anticoagulant use, we do not have access to the detailed physician notes for these patients. Therefore, in this dataset we cannot distinguish between prophylactic and therapeutic anticoagulant use. Since we include only patients who received an anticoagulant medication, there is potential for immortal time bias because there may be some patients who died before anticoagulant administration in the hospital. Another limitation of this study is the lack of follow‐up data for all patients. For many sites, we do not have access to follow‐up data for patients to determine 28‐day mortality status, so the mortality rates may be skewed towards the sites of the study where this outcome data is most available. Given the limitations of the dataset, we are unable to fully examine how differential outcomes associated with unfractionated heparin and enoxaparin may be associated with the pathogenesis of COVID‐19. There are also differences in the FDA drug labels for unfractionated heparin, enoxaparin, and other forms of low molecular weight heparin, which can lead to differences in real‐world patterns of prescription.^13^, ^14^ For example, patients with active kidney disease are contraindicated for higher doses of enoxaparin. However, unfractionated heparin does not require any dose modifications for patients with active kidney disease, so there may be a preference for this medication among this cohort of patients. Although these biases in prescription patterns are partially controlled for by the propensity score matching algorithm, there may be some additional unobserved confounding factors which are not taken into consideration. Finally, we note a disparity in patient mortality on discharge from the hospital in comparison with patient mortality at 28 days following initial admission to the hospital. Given the ongoing COVID‐19 pandemic and resultant strain on the healthcare system there is a sparsity of information available for 28 day mortality due to difficulties in reporting. Despite this relative deficiency in data, we retain analyses relying on 28 day mortality so as to not introduce a bias towards patient survival which may result from taking into account only patient mortality on discharge.

There are numerous follow‐up analyses which may be inspired from this study. For example, the biological mechanisms underlying the associations between different types of anticoagulants and clinical outcomes remain to be explored. As more data becomes available, we may investigate differential patient outcomes for other variants of low molecular weight heparin beyond enoxaparin. Similar comparative analyses may be undertaken for other COVID‐19 treatment options beyond anticoagulants, such as supplemental oxygenation methods. Important insights may also be gained from studying how varying dosing patterns of anticoagulant administration and indications driving anticoagulant administration relate to differential patient outcomes. In addition, a number of studies have been analyzing the association between race/ethnicity and clinical outcomes in COVID‐19.^15^, ^16^ The finding from this study that there are race‐associated differences in the administration of the anticoagulants enoxaparin and unfractionated heparin warrants further analyses into the associations between patients' race/ethnicity, comorbidities, and administration of medications in managing COVID‐19. Overall, this study demonstrates the utility of the SCCM VIRUS data registry for analyzing diverse research questions related to therapeutics for severe COVID‐19 patients.^5^

## CONFLICT OF INTERESTS

The authors from nference have financial interests in the company. ADB is a consultant for Abbvie, is on scientific advisory boards for nference and Zentalis, and is founder and President of Splissen therapeutics.

## ETHICS STATEMENT

The VIRUS registry was granted exempt status for human subjects research by the institutional review board at the Mayo Clinic (IRB:20‐002610). The ClinicalTrials.gov number is NCT04323787 (https://clinicaltrials.gov/ct2/show/NCT04323787). Each study site submitted a proposal to their local review boards for approval and signed a data use agreement before being granted permission to extract and enter deidentified data into the registry.

## Supporting information

Supporting information.

Supporting information.

## Data Availability

Deidentified data will be made available for research and qualitative studies purposes with appropriate approvals from SCCM after the study completion in December 2022. Reasonable requests may be made to SCCM (discovery@sccm.org) and to the corresponding author (venky@nference.net).
