# Supplementary material for: Healthcare disparities among anticoagulation therapies for severe COVID‐19 patients in the multi‐site VIRUS registry
Source: J Med Virol. 2021 Mar 30;93(7):4303–18. doi: 10.1002/jmv.26918 (PMC8013987; doi:10.1002/jmv.26918)
Supplement: Supplementary file 1 — Supporting information. [file JMV-93-4303-s001.pdf]

Supplementary Material

Supplementary Table S1. Frequency of administration of different anticoagulants.

**Anticoagulant**	**Patient Count**
Enoxaparin	2175
Unfractionated Heparin	1248
Low Molecular Weight Heparin	987
Apixaban	233
Other	210
Rivaroxaban	64
Vitamin K antagonist	34
Edoxaban	27
Argatroban	17
Fondaparinux	13
Dabigatran	12
Bivalirudin	8
TPA/RTPA	8

Supplementary Table S2: Diagnostic criteria for complications listed in the VIRUS registry questionnaire.

Acute Kidney Injury	Must meet stage 1 AKI criteria or higher as defined by 2012 Kidney Disease: Improving Global Outcomes (KDIGO) Clinical Practice Guideline for Acute Kidney Injury (AKI)<br><br>Stage 1 Criteria:<br>1) serum Cr increases by 1.5-1.9 above baseline or >= 0.3mg/dl increase in serum Cr.<br>2) Urine output < 0.5 ml/Kg/hour for 6-12 hours
Congestive Heart Failure	Heart Failure is a complex clinical syndrome resulting from any structural or functional cardiac disorder that impairs the ability of the ventricle to fill with or eject blood. It is characterized by dyspnea and fatigue in the medical history and edema, rales on physical examination.

	New York Heart Association (NYHA) Classification - The Stages of Heart Failure:<br>Class I - No symptoms and no limitation in ordinary physical activity, e.g. shortness of breath when walking, climbing stairs etc.<br>Class II - Mild symptoms (mild shortness of breath and/or angina) and slight limitation during ordinary activity.<br>Class III - Marked limitation in activity due to symptoms, even during less-than-ordinary activity, e.g. walking short distances (20—100 m). Comfortable only at rest.<br>Class IV - Severe limitations. Experiences symptoms even while at rest. Mostly bed bound patients.

Supplementary Table S3. Data completeness among the 5,065 patients with anticoagulant information available.

**Covariate**	**Number of patients with data available**
Total Patients	5065
Demographics<br>- Age<br>- Sex<br>- Race<br>- Ethnicity (Hispanic/non-Hispanic)	 4993 (99%)<br>5065 (100%)<br>5051 (100%)<br>5047 (100%)
Comorbidities	4681 (92%)
Admission diagnoses	4985 (98%)
Oxygenation methods	4949 (98%)
Evidence of infiltrates (X-ray or CT scan)	4058 (80%)

Supplementary Table S4 – Collaborative Co-authors List:

Argentina
Hospital Universitario Austral: Ana Julieta Herrera

Belgium
Centre Hospitalier Jolimont: Jean-Baptiste Mesland, Pierre Henin, Hélène Petre, Isabelle Buelens, Anne-Catherine Gerard
The Brugmann University Hospital, Bruxelles: Philippe Clevenbergh

Bolvia
Clinica Los Olivos: Rolando Claure-Del Granado, Jose A. Mercado, Esdenka Vega-Terrazas, Maria F. Iturricha-Caceres

Bosnia and Herzegovina
University Clinical Hospital, Mostar: Dragana Markotić, Ivana Bošnjak
University Clinical Centre of the Republic of Srpska, Banja Luka: Pedja Kovacevic

Columbia
Clinica Medical SAS: Oscar Y Gavidia, Felipe Pachon, Yeimy A Sanchez

Croatia
Clinical Hospital Center Rijeka: Danijel Knežević

Egypt
Helwan University: Mohamed El Kassas, Mohamed Badr, Ahmed Tawheed, Ahmed Tawheed, Hend Yahia

Hondurus
CEMESA Hospital: Sierra-Hoffman, Fernando Valerio, Oscar Diaz
Honduras Medical Center: Jose Luis Ramos Coello, Guillermo Perez, Ana Karen Vallecillo Lizardo, Gabina María Reyes Guillen, Helin Archaga Soto

Hungary
Uzsoki Teaching Hospital: Csaba Kopitkó, Ágnes Bencze, István Méhész, MD, Zsófia Gerendai

India
ACSR Govt. Medical College and Hospital: Neethi Chandra
Jawaharlal Institute of Postgraduate Medical Education and Research: Anusha Cherian, Sreejith Parameswaran, Magesh Parthiban, Menu Priya A.
KLEs Dr. Prabhakar Kore Hospital & MRC: Madhav Prabhu, Vishal Jakati
Maulana Azad Medical College and Lok Nayak Hospital: Mradul Kumar Daga, Munisha Agarwal, Ishan Rohtagi
Rising Medicare Hospital: Giri Deepak Ramgir

Japan

Center Hospital of the National Center for Global Health and Medicine: Wataru Matsuda, Reina Suzuki

Hiroshima University: Michihito Kyo

Sapporo City General Hospital: Yuki Itagaki, Akira Kodate, Reina Suzuki, Akira Kodate,Yuki Takahashi, Koyo Moriki

Tokyo Medical and Dental University: Hidenobu Shigemitsu, Yuka Mishima, Nobuyuki Nosaka, Michio Nagashima

Mexico

Centenario Hospital Miguel Hidalgo: Mariana Janeth Hermosillo Ulloa

Pakistan

Nishtar Hospital Multan: Muhammad H Khan, Muhammad Tayyeb

The Aga Khan University Hospital: Sidra Ishaque, Ali Faisal Saleem, Naved Rahman Siddiqui, Salima Sherali, Yasmin Hashwani, ShafiaI Shaque

Peru

Cayetano Heredia National Hospital: Juan Carrasco

Puerto Rico

San Juan City Hospital: Ricardo Alan Hernandez Castillo, Hector Omar Collazo Santiago

Hospital Auxilio Mutuo: Ricardo Alan Hernandez, Héctor Collazo Santiago, Héctor Collazo Santiago

Russia

Kuban State Medical University with affiliation Territorial Hospital #2: Igor Borisovich Zabolotskikh, Konstantin Dmitrievich Zybin, Sergey Vasilevich Sinkov, Tatiana Sergeevna Musaeva

Saudi Arabia

King Fahad Armed Forces Hospital: Razan K Alamoudi, Hassan M. AlSharif, Sarah A. Almazwaghi, Mohammed S Elsakran, Mohamed A Aid, Mouaz A Darwich, Omnia M Hagag, Salah A Ali, Alona Rocacorba, Kathrine Supeña, Efren Ray Juane, Jenalyn Medina, Jowany Baduria

King Faisal Specialist Hospital & Research Centre: Marwa Ridha Amer, Mohammed Abdullah Bawazeer, Talal I. Dahhan, Eiad Kseibi, Abid Shahzad Butt, Syed Moazzum Khurshid, Muath Rabee, Mohammed Abujazar, Razan Alghunaim, Maal Abualkhair, Abeer Turki AlFirm, Eiad Kseibi, Syed Moazzum Khurshid, Muath Rabee, Mohammed Abujazar, Razan Alghunaim

King Saud bin Abdulaziz University for Health Sciences and King Abdullah International Medical Research Center: Yaseen M Arabi, Sheryl Ann Abdukahil

King Saud University: Mohammed A Almazyad, Mohammed I Alarifi, Jara M Macarambon, Ahmad Abdullah Bukhari, Hussain A. Albahrani, Kazi N Asfina, Kaltham M Aldossary

Serbia

Clinical Centre of Vojvodina, Novi Sad: Gordana Jovanovic

University Hospital Center "Dr Dragisa Misovic-Dedinje": Predrag D Stevanovic, Dejan S Stojakov, Duska K Ignjatovic, Suzana C Bojic, Marina M Bobos, Irina B Nenadic, Milica S Zaric, Marko D Djuric, Vladimir R Djukic

Spain

Hospital Universitario La Paz: Santiago Y. Teruel, Belen C. Martin,Santiago Y. Teruel
Hospital Universitario, Universidad Autonoma de Nuevo León: Rene Rodriguez-Gutierrez, Jose Gerardo Gonzalez-Gonzalez, Alejandro Salcido-Montenegro, Adrian Camacho-Ortiz

United States of America

Advocate Children's Hospital, IL: Varsha P Gharpure, Usman Raheemi
Advocate Christ Medical Center: Kenneth W. Dodd, Nicholas Goodmanson, Kathleen Hesse,Paige Bird, Chauncey Weinert, Nathan Schoenrade, Abdulrahman Altaher, Esmael Mayar, Matthew Aronson, Tyler Cooper, Monica Logan, Brianna Miner, Gisele Papo
Albany Medical Center: Suzanne Barry, Christopher Woll, Gregory Wu, Erin Carrole, Kathryn Burke, Mustafa Mohammed
Allina Health (Abbott Northwestern Hospital, United Hospital and Mercy Hospital in Minnesota): Catherine A. St. Hill, Roman R. Melamed, David M. Tierney, Love A. Patel, Vino S. Raj,Barite U. Dawud, Narayana Mazumder, Abbey Sidebottom, Alena M. Guenther, Benjamin D. Krehbiel, Nova J. Schmitz, Stacy L. Jepsen
AnMed Health: Abhijit A Raval, Andrea Franks
Arkansas Children's Hospital: Katherine Irby, Ronald C. Sanders Jr., Glenda Hefley
Ascension St. Mary's Hospital: Jennifer M. Jarvis
Ascension St.Vincent Hospital Indianapolis: Anmol Kharbanda, Sunil Jhajhria, Zachary Fyffe
Ascension/St. Thomas Research Institute West Campus: Bethany Alicie
Augusta Health: Andrew S. Moyer, George M. Verghese
Augusta University Medical Center: Andrea Sikora Newsome, Christy C. Forehand, Rebecca Bruning, Timothy W. Jones
Aultman Hospital: Moldovan Sabov
Banner University Medical Center-Tucson: Jarrod M Mosier, Karen Lutrick, Beth Salvagio Campbell, Cathleen Wilson, Patrick Rivers, Jonathan Brinks, Mokenge Ndiva Mongoh, Boris Gilson
Baylor College of Medicine, Baylor St. Lukes Medical Center: Christopher M Howard, Cameron McBride, Jocelyn Abraham, Orlando Garner, Katherine Richards, Keegan Collins, Preethi Antony, Sindhu Mathew
Baylor Scott & White Health: Valerie C. Danesh, Gueorgui Dubrocq, Amber L. Davis, Marissa J Hammers, ill M. McGahey, Amanda C. Farris, Elisa Priest, Robyn Korsmo, Lorie Fares, Kathy Skiles, Susan M. Shor, Kenya Burns, Corrie A Dowell, Gabriela “Hope” Gonzales, Melody Flores, Lindsay Newman, Debora A Wilk, Jason Ettlinger, Jaccallene Bomar, Himani Darji, Alejandro Arroliga, Alejandro C Arroliga, Corrie A. Dowell, Gabriela Hope Conzales, Melody Flores, Lindsay Newman, Debora A. Wilk, Jason Ettlinger, Himani Darji, Jaccallene Bomar
Beth Israel Deaconess Medical Center: Valerie M. Banner-Goodspeed, Somnath Bose, Lauren E. Kelly, Melisa Joseph, Marie McGourty, Krystal Capers, Benjamin Hoenig, Maria C. Karamourtopoulos, Anica C. Law, Elias N. Baedorf Kassis
Boston University School of Medicine, Boston, MA: Allan J. Walkey, Sushrut S. Waikar, Michael A. Garcia, Mia Colona, Zoe Kibbelaar, Michael Leong, Daniel Wallman, Kanupriya Soni, Jennifer Maccarone, Joshua Gilman, Ycar Devis, Joseph Chung, Munizay Paracha, David N. Lumelsky, Madeline DiLorenzo, Najla Abdurrahman, Shelsey Johnson
Brooke Army Medical Center: Maj Andrew M. Hersh, CPT Stephanie L Wachs, Brittany S. Swigger, CPT Stephanie L Wachs, Capt Lauren A. Sattler, Capt Michael N. Moulton

Buffalo General Medical Center, Millard Fillmore Suburban Hospital and Oishei Children's Hospital: Kimberly Zammit, Patrick, J, McGrath, William, Loeffler,Maya, R, Chilbert
Cardinal Glennon Children's Hospital: Aaron S. Miller, Edwin L. Anderson, Rosemary Nagy, Ravali R. Inja
Cedars Sinai Medical Center: Pooja A. Nawathe, Isabel Pedraza, Jennifer Tsing, Karen Carr, Anila Chaudhary, Kathleen Guglielmino
Chambersburg Hospital: Raghavendra Tirupathi, Alymer Tang, Arshad Safi, Cindy Green, Jackie Newell
Children's Hospital Colorado, University of Colorado Anschutz Medical Campus: Katja M. Gist, Imran A Sayed, John Brinton, Larisa Strom
CHRISTUS Spohn Hospital Corpus Christi - Shoreline: Joshua J. White, Shani B. Italiya, Salim Surani, Lynn Carrasco
Clements University Hospital at UT Southwestern Medical Center: Sreekanth Cheruku, Farzin Ahmed, Christopher Deonarine, Ashley Jones, Mohammad-Ali Shaikh, David Preston, Jeanette Chin
Cox Medical Center Springfield: Steven K. Daugherty, Sam Atkinson, Kelly Shrimpton
Detar Family Medicine residency: Sidney Ontai, Brian Contreras, MD, Uzoma Obinwanko, Nneka Amamasi, Amir Sharafi
DeTar Hospital: Salim Surani, Sidney C. Ontai, Brian Contreras, Daniel Handayan, Jeremy George, Janet Le, Aniruddha Gollapalli, Iqra Qureshi
DeTar/Texas A&M Family Medicine Residency: Harish Chandna, Sidney C. Ontai, Amirhossein Sharafi, Iqra Qureshi, MD, Hasan Yasin
George Washington University: David P. Yamane, Ivy Benjenk, Nivedita Prasanna
Hassenfeld Children's Hospital at NYU Langone: Heda R. Dapul, Sourabh Verma, Alan Salas, Ariel Daube, Michelle Korn, Michelle Ramirez, Logi Rajagopalan, Laura Santos
Howard University Hospital Washington: Norma Smalls Mantey
Jacobs Medical Center UC San Diego Health – La Jolla: Atul Malhotra, Abdurrahman Husain, Qais Zawaydeh
Johns Hopkins School of Medicine: J.H. Steuernagle
JPS Health Network: Steven Q. Davis, Valentina Jovic, Valentina Jovic, Max Masuda, Amanda Hayes
KCPCRU at Norton Children's Hospital Louisville: Melissa Thomas, Sarah Morris, Jennifer Nason
LifeBridge Health/Sinai and Northwest Hospitals: Jaime Simon Barnes, Namrata Nag
Lincoln Medical Center: Manoj K Gupta, Franscene E. Oulds, Akshay Nandavar
Loyola University Medical Center: Yuk Ming Liu, Sarah Zavala, Sarah Zavala, Esther Shim
M Health-Fairview, University of Minnesota: Ronald A. Reilkoff, Julia A. Heneghan, Sarah Eichen, Lexie Goertzen, Scott Rajala, Ghislaine Feussom, Ben Tang
Mayo Clinic Arizona: Rodrigo Cartin-Ceba, Ayan Sen, Amanda Palacios, Giyth M. Mahdi
Mayo Clinic Rochester: Rahul Kashyap, Ognjen Gajic, Aysun Tekin, Amos Lal, John C. O'Horo, Neha N. Deo, Mayank Sharma, Shahraz Qamar
Mayo Clinic, Florida: Devang Sanghavi, Pramod Guru, Karthik Gnanapandithan, Hollie Saunders, Zachary Fleissner, Juan Garcia , Alejandra Yu Lee Mateus, Siva Naga Yarrarapu
Mayo Clinic, Mankato: Syed Anjum Khan, Juan Pablo Domecq, Nitesh Kumar Jain, Thoyaja Koritala
Mayo Clinic, Eau Claire: Abigail T. La Nou, Marija Bogojevic
Medical Center Navicent Health: Amy B. Christie, Dennis W. Ashley, Rajani Adiga
Medical College of Wisconsin: Rahul S Nanchal, Paul A Bergl, Jennifer L Peterson
Mercy Hospital and Medical Center, Chicago: Travis Yamanaka, Nicholas A. Barreras, Michael Markos, Anita Fareeduddin, Rohan Mehta
Mercy Hospital, Saint Louis: Chakradhar Venkata, Miriam Engemann, Annamarie Mantese

Nazareth Hospital Trinity Health Mid-Atlantic Philadelphia: Racheal Park
New Hanover Regional Medical Center: Nasar A Siddiqi, Lesly Jurado, Lindsey Tincher, Carolyn Brown
OSF Saint Francis Medical Center: Bhagat S. Aulakh, Sandeep Tripathi, Jennifer A. Bandy, Lisa M. Kreps, Dawn R. Bollinger, Jennifer A. Bandy
OU Medical Center: Neha Gupta, Brent R Brown, Tracy L Jones, Kassidy Malone, Lauren A Sinko, Amy B Harrell, Shonda C Ayers, Lisa M Settle, Taylor J Sears
Parkview Health System, Fort Wayne: Roger Scott Stienecker, Andre G. Melendez, Tressa A. Brunner, Sue M Budzon, Jessica L. Heffernan, Janelle M. Souder, Tracy L. Miller, Andrea G. Maisonneuve
Ridgecrest Regional Hospital: Victoria Schauf
Roper St. Francis Healthcare Charleston: Sara Utley, Holly Balcer
Saint Alphonsus Regional Medical Center: Kerry P. J. Pulver, Jennifer Yehle, Alicia Weeks, Terra Inman
Saint Luke's Hospital: Majdi Hamarshi, Jeannette Ploetz, Nick Bennett, Kyle Klindworth, Moustafa Younis, Adham Mohamed
Samaritan Health Services: Brian L. Delmonaco, Anthony Franklin, Mitchell Heath
Santa Barbara Cottage Hospital: Diane Barkas
Sarasota Memorial Hospital: Antonia L. Vilella, Sara B. Kutner, Kacie Clark, Danielle Moore
Seattle Children's Hospital: Shina Menon, John K McGuire, Deana Rich
St. Joseph Mercy Ann Arbor, Ann Arbor: Harry L. Anderson, III, Dixy Rajkumar, Ali Abunayla, Jerrilyn Heiter
St. Joseph's Candler Health System: Howard A. Zaren, Stephanie J. Smith, Grant C. Lewis, Lauren Seames, Cheryl Farlow, Judy Miller, Gloria Broadstreet
St.Agnes Hospital: Anthony Martinez, Micheal Allison, Aniket Mittal, Rafael Ruiz, Aleta Skaanland, Robert Ross
St.Mary Medical Center, Langhorne: Umang Patel, Jordesha Hodge, KrunalKumar Patel, Shivani Dalal, Himanshu Kavani, Sam Joseph
Stamford Health: Michael A. Bernstein, Ian K. Goff, Matthew Naftilan, Amal Mathew, Deborah Williams, Sue Murdock, RN, Maryanne Ducey, Kerianne Nelson
Stanford Hospital and Clinics: Paul K Mohabir, Connor G O'Brien, Komal Dasani
SUNY Upstate Medical University: William Marx, Ioana Amzuta, Asad J. Choudhry, Mohammad T. Azam
Temple University: Daniel A Salerno
The Children's Hospital at OU Medicine: Neha Gupta, Tracy L Jones, Shonda C Ayers, Amy B Harrell, Dr. Brent R Brown
The Children's Hospital of San Antonio, Baylor College of Medicine: Utpal S. Bhalala, Joshua Kuehne, Melinda Garcia, Morgan Beebe, Heather Herrera
The Queen's Medical Center: Chris Fiack, Stephanie Guo, May Vawer, Beth Blackburn
Thomas Jefferson University Hospital: Katherine A. Belden, Michael Baram, Devin M. Weber, Rosalie DePaola, Yuwei Xia, Hudson Carter, Aaron Tolley
Truman Medical Centers: Mark Steele, Laurie Kemble
Tulane University Medical Center and University Medical Center New Orleans: Joshua L. Denson, A. Scott Gillet, Margo Brown, Rachael Stevens, Andrew Wetherbie, Kevin Tea, Mathew Moore
UC San Diego Medical Center – Hillcrest: Abdurrahman Husain, Atul Malhotra, Qais Zawaydeh
UC San Diego Medical Center -Jacobs: Atul Malhotra, Abdurrahman Husain, Qais Zawaydeh
UNC Medical Center: Benjamin J Sines, Thomas J Bice

University Medical Center (University Medical Center of Southern Nevada Las Vegas): Rajany V. Dy, Alfredo Iardino, Jill Sharma, Richard Czieki, Julia Christopher, Ryan Lacey, Marwan Mashina,, Kushal Patel
University of Alabama at Birmingham: Erica C. Bjornstad, Nancy M. Tofil, Scott House, Isabella Aldana
University of Arkansas for Medical Sciences: Nikhil K. Meena, Jose D. Caceres, Nikhil K Meena, Sarenthia M. Epps, Harmeen Goraya, Kelsey R. Besett, MD, Ryan James, Lana Y. Abusalem, Akash K. Patel, Lana S Hasan
University of Chicago: Casey W Stulce, Grace Chong, Ahmeneh Ghavam, Anoop Mayampurath
University of Cincinnati: Dina Gomaa B.S., Michael Goodman, Devin Wakefield, Anthony Spuzzillo, John O. Shinn II
University of Colorado Hospital: Robert MacLaren
University of Florida Health Shands Hospital: Azra Bihorac, Tezcan Ozrazgat Baslanti, George Omalay, Haleh Hashemighouchani, Julie S. Cupka, Matthew M Ruppert
University of Iowa Carver College of Medicine: Patrick W. McGonagill, Colette Galet, Janice Hubbard, David Wang, Lauren Allan, Aditya Badheka, Madhuradhar Chegondi
University of Kansas Medical Center: Usman Nazir, Garrett Rampon, Jake Riggle, Nathan Dismang
University of Louisville Hospital: Ozan Akca, Rainer Lenhardt, Rodrigo S. Cavallazzi, Ann Jerde, Alexa Black, Allison Polidori, Haily Griffey, Justin Winkler, Thomas Brenzel
University of Miami Miller School of Medicine: Roger A. Alvarez, Amarilys Alarcon-Calderon, Marie Anne Sosa, Sunita K. Mahabir, Mausam J. Patel
University of Michigan Health System: Pauline Park, Andrew Admon, Sinan Hanna, Rishi Chanderraj, Maria Pliakas, Ann Wolski, Jennifer Cirino
University of Missouri, Columbia: Dima Dandachi, Hariharan Regunath, Maraya N. Camazine, Grant. E. Geiger, Abdoulie O. Njai, Baraa M. Saad
University of Utah Health: Joseph E. Tonna, Nicholas M. Levin, Kayte Suslavich, Rachel Tsolinas, Zachary T. Fica, Chloe R. Skidmore
University of Vermont Larner College of Medicine: Renee D. Stapleton, Anne E. Dixon, Olivia Johnson, Sara S. Ardren, Stephanie Burns, Anna Raymond, Erika Gonyaw, Kevin Hodgdon, Chloe Housenger, Benjamin Lin, Karen McQuesten, Heidi Pecott-Grimm, Julie Sweet, Sebastian Ventrone
Valleywise Health (formerly Maricopa Medical Center): Murtaza Akhter, Rania Abdul Rahman, Mary Mulrow
Vanderbilt University Medical Center: Erin M. Wilfong, Kelsi Vela
Wake Forest University School of Medicine; Wake Forest Baptist Health Network: Ashish K. Khanna, Lynne Harris, Bruce Cusson, Jacob Fowler, David Vaneenenaam, Glen McKinney, Imoh Udoh, Kathleen Johnson
Washington University School of Medicine and Barnes-Jewish Hospital: Patrick G. Lyons, Andrew P Michelson, Sara S. Haluf, Lauren M. Lynch, Nguyet M. Nguyen, Aaron Steinberg
West Virginia University Morgantown: Ankit Sakhuja
William S. Middleton Memorial VA Hospital Madison: Nicholas A Braus
Yale New Haven Health New Haven: Kevin N Sheth, Abdalla A Ammar
