# Supplementary material for: Healthcare disparities among anticoagulation therapies for severe COVID‐19 patients in the multi‐site VIRUS registry
Source: J Med Virol. 2021 Mar 30;93(7):4303–18. doi: 10.1002/jmv.26918 (PMC8013987; doi:10.1002/jmv.26918)
Supplement: Supplementary file 2 — Supporting information. [file JMV-93-4303-s002.docx]

**Collaborative Co-authors List for Appendix: (anticoagulation therapies, Kirkup et,al)**

| **Argentina** |
| --- |
| **Hospital Universitario Austral:** Ana Julieta Herrera |
|  |
| **Belgium** |
| **Centre Hospitalier Jolimont:** Jean-Baptiste Mesland, Pierre Henin, Hélène Petre, Isabelle Buelens, Anne-Catherine Gerard |
| **The Brugmann University Hospital, Bruxelles:** Philippe Clevenbergh |
|  |
| **Bolvia** |
| **Clinica Los Olivos:** Rolando Claure-Del Granado, Jose A. Mercado, Esdenka Vega-Terrazas, Maria F. Iturricha-Caceres |
|  |
| **Bosnia and Herzegovina** |
| **University Clinical Hospital, Mostar:** Dragana Markotić, Ivana Bošnjak |
| **University Clinical Centre of the Republic of Srpska, Banja Luka:** Pedja Kovacevic |
|  |
| **Columbia** |
| **Clinica Medical SAS:** Oscar Y Gavidia, Felipe Pachon, Yeimy A Sanchez |
|  |
| **Croatia** |
| **Clinical Hospital Center Rijeka:** Danijel Knežević |
|  |
| **Egypt** |
| **Helwan University:** Mohamed El Kassas, Mohamed Badr, Ahmed Tawheed, Ahmed Tawheed, Hend Yahia |
|  |
| **Hondurus** |
| **CEMESA Hospital:** Sierra-Hoffman, Fernando Valerio, Oscar Diaz |
| **Honduras Medical Center:** Jose Luis Ramos Coello, Guillermo Perez, Ana Karen Vallecillo Lizardo, Gabina María Reyes Guillen, Helin Archaga Soto |
|  |
| **Hungary** |
| **Uzsoki Teaching Hospital:** Csaba Kopitkó, Ágnes Bencze, István Méhész, MD, Zsófia Gerendai |
|  |
| **India** |
| **ACSR Govt. Medical College and Hospital:** Neethi Chandra |
| **Jawaharlal Institute of Postgraduate Medical Education and Research:** Anusha Cherian, Sreejith Parameswaran, Magesh Parthiban, Menu Priya A**.** |
| **KLEs Dr. Prabhakar Kore Hospital & MRC:** Madhav Prabhu, Vishal Jakati |
| **Maulana Azad Medical College and Lok Nayak Hospital:** Mradul Kumar Daga, Munisha Agarwal, Ishan Rohtagi |
| **Rising Medicare Hospital:** Giri Deepak Ramgir |
|  |
| **Japan** |
| **Center Hospital of the National Center for Global Health and Medicine:** Wataru Matsuda, Reina Suzuki |
| **Hiroshima University:** Michihito Kyo |
| **Sapporo City General Hospital:** Yuki Itagaki, Akira Kodate, Reina Suzuki, Akira Kodate,Yuki Takahashi, Koyo Moriki |
| **Tokyo Medical and Dental University:** Hidenobu Shigemitsu, Yuka Mishima, Nobuyuki Nosaka, Michio Nagashima |
|  |
| **Mexico** |
| **Centenario Hospital Miguel Hidalgo:** Mariana Janeth Hermosillo Ulloa |
|  |
| **Pakistan** |
| **Nishtar Hospital Multan:** Muhammad H Khan, Muhammad Tayyeb |
| **The Aga Khan University Hospital:** Sidra Ishaque, Ali Faisal Saleem, Naved Rahman Siddiqui, Salima Sherali, Yasmin Hashwani, ShafiaI Shaque |
|  |
| **Peru** |
| **Cayetano Heredia National Hospital:** Juan Carrasco |
|  |
| **Puerto Rico** |
| **San Juan City Hospital:** Ricardo Alan Hernandez Castillo, Hector Omar Collazo Santiago |
| **Hospital Auxilio Mutuo:** Ricardo Alan Hernandez, Héctor Collazo Santiago, Héctor Collazo Santiago |
|  |
| **Russia** |
| **Kuban State Medical University with affiliation Territorial Hospital #2:** Igor Borisovich Zabolotskikh, Konstantin Dmitrievich Zybin, Sergey Vasilevich Sinkov, Tatiana Sergeevna Musaeva |
|  |
| **Saudi Arabia** |
| **King Fahad Armed Forces Hospital:** Razan K Alamoudi, Hassan M. AlSharif, Sarah A. Almazwaghi, Mohammed S Elsakran, Mohamed A Aid, Mouaz A Darwich, Omnia M Hagag, Salah A Ali, Alona Rocacorba, Kathrine Supeña, Efren Ray Juane, Jenalyn Medina, Jowany Baduria |
| **King Faisal Specialist Hospital & Research Centre:** Marwa Ridha Amer, Mohammed Abdullah Bawazeer, Talal I. Dahhan, Eiad Kseibi, Abid Shahzad Butt, Syed Moazzum Khurshid, Muath Rabee, Mohammed Abujazar, Razan Alghunaim, Maal Abualkhair, Abeer Turki AlFirm, Eiad Kseibi, Syed Moazzum Khurshid, Muath Rabee, Mohammed Abujazar, Razan Alghunaim |
| **King Saud bin Abdulaziz University for Health Sciences and King Abdullah International Medical Research Center:** Yaseen M Arabi, Sheryl Ann Abdukahil |
| **King Saud University:** Mohammed A Almazyad, Mohammed I Alarifi, Jara M Macarambon, Ahmad Abdullah Bukhari, Hussain A. Albahrani, Kazi N Asfina, Kaltham M Aldossary |
|  |
| **Serbia** |
| **Clinical Centre of Vojvodina, Novi Sad:** Gordana Jovanovic |
| **University Hospital Center "Dr Dragisa Misovic-Dedinje":** Predrag D Stevanovic, Dejan S Stojakov, Duska K Ignjatovic, Suzana C Bojic, Marina M Bobos, Irina B Nenadic, Milica S Zaric, Marko D Djuric, Vladimir R Djukic |
|  |
| **Spain** |
| **Hospital Universitario La Paz:** Santiago Y. Teruel, Belen C. Martin,Santiago Y. Teruel |
| **Hospital Universitario, Universidad Autonoma de Nuevo León:** Rene Rodriguez-Gutierrez, Jose Gerardo Gonzalez-Gonzalez, Alejandro Salcido-Montenegro, Adrian Camacho-Ortiz |
|  |
| **United States of America** |
| **Advocate Children's Hospital, IL:** Varsha P Gharpure, Usman Raheemi |
| **Advocate Christ Medical Center:** Kenneth W. Dodd, Nicholas Goodmanson, Kathleen Hesse,Paige Bird, Chauncey Weinert, Nathan Schoenrade, Abdulrahman Altaher, Esmael Mayar, Matthew Aronson, Tyler Cooper, Monica Logan, Brianna Miner, Gisele Papo |
| **Albany Medical Center:** Suzanne Barry, Christopher Woll, Gregory Wu, Erin Carrole, Kathryn Burke, Mustafa Mohammed |
| **Allina Health (Abbott Northwestern Hospital, United Hospital and Mercy Hospital in Minnesota):** Catherine A. St. Hill, Roman R. Melamed, David M. Tierney, Love A. Patel, Vino S. Raj,Barite U. Dawud, Narayana Mazumder, Abbey Sidebottom, Alena M. Guenther, Benjamin D. Krehbiel, Nova J. Schmitz, Stacy L. Jepsen |
| **AnMed Health:** Abhijit A Raval, Andrea Franks |
| **Arkansas Children's Hospital:** Katherine Irby, Ronald C. Sanders Jr., Glenda Hefley |
| **Ascension St. Mary's Hospital:** Jennifer M. Jarvis |
| **Ascension St.Vincent Hospital Indianapolis:** Anmol Kharbanda, Sunil Jhajhria, Zachary Fyffe |
| **Ascension/St. Thomas Research Institute West Campus:** Bethany Alicie |
| **Augusta Health:** Andrew S. Moyer, George M. Verghese |
| **Augusta University Medical Center:** Andrea Sikora Newsome, Christy C. Forehand, Rebecca Bruning, Timothy W. Jones |
| **Aultman Hospital:** Moldovan Sabov |
| **Banner University Medical Center-Tucson:** Jarrod M Mosier, Karen Lutrick, Beth Salvagio Campbell, Cathleen Wilson, Patrick Rivers, Jonathan Brinks, Mokenge Ndiva Mongoh, Boris Gilson |
| **Baylor College of Medicine, Baylor St. Lukes Medical Center:** Christopher M Howard, Cameron McBride, Jocelyn Abraham, Orlando Garner, Katherine Richards, Keegan Collins, Preethi Antony, Sindhu Mathew |
| **Baylor Scott & White Health:** Valerie C. Danesh, Gueorgui Dubrocq, Amber L. Davis, Marissa J Hammers, ill M. McGahey, Amanda C. Farris, Elisa Priest, Robyn Korsmo, Lorie Fares, Kathy Skiles, Susan M. Shor, Kenya Burns, Corrie A Dowell, Gabriela “Hope” Gonzales, Melody Flores, Lindsay Newman, Debora A Wilk, Jason Ettlinger, Jaccallene Bomar, Himani Darji, Alejandro Arroliga, Alejandro C Arroliga, Corrie A. Dowell, Gabriela Hope Conzales, Melody Flores, Lindsay Newman, Debora A. Wilk, Jason Ettlinger, Himani Darji, Jaccallene Bomar |
| **Beth Israel Deaconess Medical Center:** Valerie M. Banner-Goodspeed, Somnath Bose, Lauren E. Kelly, Melisa Joseph, Marie McGourty, Krystal Capers, Benjamin Hoenig, Maria C. Karamourtopoulos, Anica C. Law, Elias N. Baedorf Kassis |
| **Boston University School of Medicine, Boston, MA:** Allan J. Walkey, Sushrut S. Waikar, Michael A. Garcia, Mia Colona, Zoe Kibbelaar, Michael Leong, Daniel Wallman, Kanupriya Soni, Jennifer Maccarone, Joshua Gilman, Ycar Devis, Joseph Chung, Munizay Paracha, David N. Lumelsky, Madeline DiLorenzo, Najla Abdurrahman, Shelsey Johnson |
| **Brooke Army Medical Center:** Maj Andrew M. Hersh, CPT Stephanie L Wachs, Brittany S. Swigger, CPT Stephanie L Wachs, Capt Lauren A. Sattler, Capt Michael N. Moulton |
| **Buffalo General Medical Center, Millard Fillmore Suburban Hospital and Oishei Children’s Hospital:** Kimberly Zammit, Patrick, J, McGrath, William, Loeffler,Maya, R, Chilbert |
| **Cardinal Glennon Children's Hospital:** Aaron S. Miller, Edwin L. Anderson, Rosemary Nagy, Ravali R. Inja |
| **Cedars Sinai Medical Center:** Pooja A. Nawathe, Isabel Pedraza, Jennifer Tsing, Karen Carr, Anila Chaudhary, Kathleen Guglielmino |
| **Chambersburg Hospital:** Raghavendra Tirupathi, Alymer Tang, Arshad Safi, Cindy Green, Jackie Newell |
| **Children's Hospital Colorado, University of Colorado Anschutz Medical Campus:** Katja M. Gist, Imran A Sayed, John Brinton, Larisa Strom |
| **CHRISTUS Spohn Hospital Corpus Christi - Shoreline:** Joshua J. White, Shani B. Italiya, Salim Surani, Lynn Carrasco |
| **Clements University Hospital at UT Southwestern Medical Center:** Sreekanth Cheruku, Farzin Ahmed, Christopher Deonarine, Ashley Jones, Mohammad-Ali Shaikh, David Preston, Jeanette Chin |
| **Cox Medical Center Springfield:** Steven K. Daugherty, Sam Atkinson, Kelly Shrimpton |
| **Detar Family Medicine residency:** Sidney Ontai, Brian Contreras, MD, Uzoma Obinwanko, Nneka Amamasi, Amir Sharafi |
| **DeTar Hospital:** Salim Surani, Sidney C. Ontai, Brian Contreras, Daniel Handayan, Jeremy George, Janet Le, Aniruddha Gollapalli, Iqra Qureshi |
| **DeTar/Texas A&M Family Medicine Residency:** Harish Chandna, Sidney C. Ontai, Amirhossein Sharafi, Iqra Qureshi, MD, Hasan Yasin |
| **George Washington University:** David P. Yamane, Ivy Benjenk, Nivedita Prasanna |
| **Hassenfeld Children's Hospital at NYU Langone:** Heda R. Dapul, Sourabh Verma, Alan Salas, Ariel Daube, Michelle Korn, Michelle Ramirez, Logi Rajagopalan, Laura Santos |
| **Howard University Hospital Washington:** Norma Smalls Mantey |
| **Jacobs Medical Center UC San Diego Health – La Jolla:** Atul Malhotra, Abdurrahman Husain, Qais Zawaydeh |
| **Johns Hopkins School of Medicine:** J.H. Steuernagle |
| **JPS Health Network:** Steven Q. Davis, Valentina Jovic, Valentina Jovic, Max Masuda, Amanda Hayes |
| **KCPCRU at Norton Children’s Hospital Louisville:** Melissa Thomas, Sarah Morris, Jennifer Nason |
| **LifeBridge Health/Sinai and Northwest Hospitals:** Jaime Simon Barnes, Namrata Nag |
| **Lincoln Medical Center:** Manoj K Gupta, Franscene E. Oulds, Akshay Nandavar |
| **Loyola University Medical Center:** Yuk Ming Liu, Sarah Zavala, Sarah Zavala, Esther Shim |
| **M Health-Fairview, University of Minnesota:** Ronald A. Reilkoff, Julia A. Heneghan, Sarah Eichen, Lexie Goertzen, Scott Rajala, Ghislaine Feussom, Ben Tang |
| **Mayo Clinic Arizona:** Rodrigo Cartin-Ceba, Ayan Sen, Amanda Palacios, Giyth M. Mahdi |
| **Mayo Clinic Rochester:** Rahul Kashyap, Ognjen Gajic, Aysun Tekin, Amos Lal, John C. O'Horo, Neha N. Deo, Mayank Sharma, Shahraz Qamar |
| **Mayo Clinic, Florida:** Devang Sanghavi, Pramod Guru, Karthik Gnanapandithan, Hollie Saunders, Zachary Fleissner, Juan Garcia , Alejandra Yu Lee Mateus, Siva Naga Yarrarapu |
| **Mayo Clinic, Mankato:** Syed Anjum Khan, Juan Pablo Domecq, Nitesh Kumar Jain, Thoyaja Koritala |
| **Mayo Clinic, Eau Claire**: Abigail T. La Nou, Marija Bogojevic |
| **Medical Center Navicent Health:** Amy B. Christie, Dennis W. Ashley, Rajani Adiga |
| **Medical College of Wisconsin:** Rahul S Nanchal, Paul A Bergl, Jennifer L Peterson |
| **Mercy Hospital and Medical Center, Chicago:** Travis Yamanaka, Nicholas A. Barreras, Michael Markos, Anita Fareeduddin, Rohan Mehta |
| **Mercy Hospital, Saint Louis:** Chakradhar Venkata, Miriam Engemann, Annamarie Mantese |
| **Nazareth Hospital Trinity Health Mid-Atlantic Philadelphia:** Racheal Park |
| **New Hanover Regional Medical Center:** Nasar A Siddiqi, Lesly Jurado, Lindsey Tincher, Carolyn Brown |
| **OSF Saint Francis Medical Center:** Bhagat S. Aulakh, Sandeep Tripathi, Jennifer A. Bandy, Lisa M. Kreps, Dawn R. Bollinger, Jennifer A. Bandy |
| **OU Medical Center:** Neha Gupta, Brent R Brown, Tracy L Jones, Kassidy Malone, Lauren A Sinko, Amy B Harrell, Shonda C Ayers, Lisa M Settle, Taylor J Sears |
| **Parkview Health System, Fort Wayne:** Roger Scott Stienecker, Andre G. Melendez, Tressa A. Brunner, Sue M Budzon, Jessica L. Heffernan, Janelle M. Souder, Tracy L. Miller, Andrea G. Maisonneuve |
| **Ridgecrest Regional Hospital:** Victoria Schauf |
| **Roper St. Francis Healthcare Charleston:** Sara Utley, Holly Balcer |
| **Saint Alphonsus Regional Medical Center:** Kerry P. J. Pulver, Jennifer Yehle, Alicia Weeks, Terra Inman |
| **Saint Luke's Hospital:** Majdi Hamarshi, Jeannette Ploetz, Nick Bennett, Kyle Klindworth, Moustafa Younis, Adham Mohamed |
| **Samaritan Health Services:** Brian L. Delmonaco, Anthony Franklin, Mitchell Heath |
| **Santa Barbara Cottage Hospital:** Diane Barkas |
| **Sarasota Memorial Hospital:** Antonia L. Vilella, Sara B. Kutner, Kacie Clark, Danielle Moore |
| **Seattle Children's Hospital:** Shina Menon, John K McGuire, Deana Rich |
| **St. Joseph Mercy Ann Arbor, Ann Arbor:** Harry L. Anderson, III, Dixy Rajkumar, Ali Abunayla, Jerrilyn Heiter |
| **St. Joseph's Candler Health System:** Howard A. Zaren, Stephanie J. Smith, Grant C. Lewis, Lauren Seames, Cheryl Farlow, Judy Miller, Gloria Broadstreet |
| **St.Agnes Hospital:** Anthony Martinez, Micheal Allison, Aniket Mittal, Rafael Ruiz, Aleta Skaanland, Robert Ross |
| **St.Mary Medical Center, Langhorne:** Umang Patel, Jordesha Hodge, KrunalKumar Patel, Shivani Dalal, Himanshu Kavani, Sam Joseph |
| **Stamford Health:** Michael A. Bernstein, Ian K. Goff, Matthew Naftilan, Amal Mathew, Deborah Williams, Sue Murdock, RN, Maryanne Ducey, Kerianne Nelson |
| **Stanford Hospital and Clinics:** Paul K Mohabir, Connor G O'Brien, Komal Dasani |
| **SUNY Upstate Medical University:** William Marx, Ioana Amzuta, Asad J. Choudhry, Mohammad T. Azam |
| **Temple University:** Daniel A Salerno |
| **The Children's Hospital at OU Medicine:** Neha Gupta, Tracy L Jones, Shonda C Ayers, Amy B Harrell, Dr. Brent R Brown |
| **The Children's Hospital of San Antonio, Baylor College of Medicine:** Utpal S. Bhalala, Joshua Kuehne, Melinda Garcia, Morgan Beebe, Heather Herrera |
| **The Queen's Medical Center:** Chris Fiack, Stephanie Guo, May Vawer, Beth Blackburn |
| **Thomas Jefferson University Hospital:** Katherine A. Belden, Michael Baram, Devin M. Weber, Rosalie DePaola, Yuwei Xia, Hudson Carter, Aaron Tolley |
| **Truman Medical Centers**: Mark Steele, Laurie Kemble |
| **Tulane University Medical Center and University Medical Center New Orleans:** Joshua L. Denson, A. Scott Gillet, Margo Brown, Rachael Stevens, Andrew Wetherbie, Kevin Tea, Mathew Moore |
| **UC San Diego Medical Center – Hillcrest:** Abdurrahman Husain, Atul Malhotra, Qais Zawaydeh |
| **UC San Diego Medical Center -Jacobs:** Atul Malhotra, Abdurrahman Husain, Qais Zawaydeh |
| **UNC Medical Center:** Benjamin J Sines, Thomas J Bice |
| **University Medical Center (University Medical Center of Southern Nevada Las Vegas):** Rajany V. Dy, Alfredo Iardino, Jill Sharma, Richard Czieki, Julia Christopher, Ryan Lacey, Marwan Mashina,, Kushal Patel |
| **University of Alabama at Birmingham:** Erica C. Bjornstad, Nancy M. Tofil, Scott House, Isabella Aldana |
| **University of Arkansas for Medical Sciences:** Nikhil K. Meena, Jose D. Caceres, Nikhil K Meena, Sarenthia M. Epps, Harmeen Goraya, Kelsey R. Besett, MD, Ryan James, Lana Y. Abusalem, Akash K. Patel, Lana S Hasan |
| **University of Chicago:** Casey W Stulce, Grace Chong, Ahmeneh Ghavam, Anoop Mayampurath |
| **University of Cincinnati:** Dina Gomaa B.S., Michael Goodman, Devin Wakefield, Anthony Spuzzillo, John O. Shinn II |
| **University of Colorado Hospital:** Robert MacLaren |
| **University of Florida Health Shands Hospital:** Azra Bihorac, Tezcan Ozrazgat Baslanti, George Omalay, Haleh Hashemighouchani, Julie S. Cupka, Matthew M Ruppert |
| **University of Iowa Carver College of Medicine:** Patrick W. McGonagill, Colette Galet, Janice Hubbard, David Wang, Lauren Allan, Aditya Badheka, Madhuradhar Chegondi |
| **University of Kansas Medical Center:** Usman Nazir, Garrett Rampon, Jake Riggle, Nathan Dismang |
| **University of Louisville Hospital:** Ozan Akca, Rainer Lenhardt, Rodrigo S. Cavallazzi, Ann Jerde, Alexa Black, Allison Polidori, Haily Griffey, Justin Winkler, Thomas Brenzel |
| **University of Miami Miller School of Medicine:** Roger A. Alvarez, Amarilys Alarcon-Calderon, Marie Anne Sosa, Sunita K. Mahabir, Mausam J. Patel |
| **University of Michigan Health System:** Pauline Park, Andrew Admon, Sinan Hanna, Rishi Chanderraj, Maria Pliakas, Ann Wolski, Jennifer Cirino |
| **University of Missouri, Columbia:** Dima Dandachi, Hariharan Regunath, Maraya N. Camazine, Grant. E. Geiger, Abdoulie O. Njai, Baraa M. Saad |
| **University of Utah Health:** Joseph E. Tonna, Nicholas M. Levin, Kayte Suslavich, Rachel Tsolinas, Zachary T. Fica, Chloe R. Skidmore |
| **University of Vermont Larner College of Medicine:** Renee D. Stapleton, Anne E. Dixon, Olivia Johnson, Sara S. Ardren, Stephanie Burns, Anna Raymond, Erika Gonyaw, Kevin Hodgdon, Chloe Housenger, Benjamin Lin, Karen McQuesten, Heidi Pecott-Grimm, Julie Sweet, Sebastian Ventrone |
| **Valleywise Health (formerly Maricopa Medical Center):** Murtaza Akhter, Rania Abdul Rahman, Mary Mulrow |
| **Vanderbilt University Medical Center:** Erin M. Wilfong, Kelsi Vela |
| **Wake Forest University School of Medicine; Wake Forest Baptist Health Network:** Ashish K. Khanna, Lynne Harris, Bruce Cusson, Jacob Fowler, David Vaneenenaam, Glen McKinney, Imoh Udoh, Kathleen Johnson |
| **Washington University School of Medicine and Barnes-Jewish Hospital:** Patrick G. Lyons, Andrew P Michelson, Sara S. Haluf, Lauren M. Lynch, Nguyet M. Nguyen, Aaron Steinberg |
| **West Virginia University Morgantown:** Ankit Sakhuja |
| **William S. Middleton Memorial VA Hospital Madison:** Nicholas A Braus |
| **Yale New Haven Health New Haven:** Kevin N Sheth, Abdalla A Ammar |
|  |
|  |
